# Microbial community structural and functional differentiation in capped thickened oil sands tailings planted with native boreal species

**DOI:** 10.3389/fmicb.2023.1168653

**Published:** 2023-07-03

**Authors:** Abdul Samad, Dani Degenhardt, Armand Séguin, Marie-Josée Morency, Patrick Gagné, Christine Martineau

**Affiliations:** ^1^Natural Resources Canada, Canadian Forest Service, Laurentian Forestry Centre, Québec City, QC, Canada; ^2^Natural Resources Canada, Canadian Forest Service, Northern Forestry Centre, Edmonton, AB, Canada

**Keywords:** oil sands tailings, reclamation, metabarcoding, metagenomics, ecological restoration, soil microbiology, boreal forest

## Abstract

The oil sands mining operations in Alberta have produced billions of m^3^ of tailings which must be reclaimed and integrated into various mine closure landforms, including terrestrial landforms. Microorganisms play a central role in nutrient cycling during the reclamation of disturbed landscapes, contributing to successful vegetation restoration and long-term sustainability. However, microbial community succession and response in reconstructed and revegetated tailings remain largely unexplored. This study aimed to monitor the structural and functional responses of microbial communities in tailings subjected to different capping and vegetation strategies over two growing seasons (GS). To achieve this, a column-based greenhouse experiment was conducted to investigate microbial communities in tailings that were capped with a layer (10 or 30 cm) of peat-mineral mix (PMM) and planted with either upland or wetland communities. DNA metabarcoding analysis of the bacterial 16S rRNA gene and fungal ITS2 region as well as shotgun metagenomics were used to asses the impact of treatments on microbial taxonomy and functions, respectively. Results showed that tailings microbial diversity and community composition changed considerably after two GS compared to baseline samples, while communities in the PMM capping layer were much more stable. Likewise, several microbial functions were significantly enriched in tailings after two GS. Interestingly, the impact of capping on bacterial communities in tailings varied depending on the plant community, leading to a higher number of differentially abundant taxa and to a decrease in Shannon diversity and evenness in the upland treatment but not in the wetland treatment. Moreover, while capping in the presence of wetland vegetation increased the energy-related metabolic functions (carbon, nitrogen, and sulfur), these functions were depleted by capping in the upland treatment. Fungi represented a small proportion of the microbial community in tailings, but the relative abundance of several taxa changed over time, while the capping treatments favored the growth of some beneficial taxa, notably the root endophyte *Serendipita*, in both upland and wetland columns. The results suggest that selecting the right combination of capping material and vegetation type may contribute to improve below-ground microbial processes and sustain plant growth in harsh environments such as oil sands tailings.

## 1. Introduction

Oil sands surface mining in northern Alberta has generated over a billion m^3^ of a waste stream called tailings stored temporarily in tailings ponds which must be reclaimed to an equivalent land capability to the pre-disturbance landscape, as directed by environmental regulations (Chalaturnyk et al., 2002; Parrott et al., 2019; Cossey et al., 2021). Fluid tailings are composed of water and solids, typically consisting of sand, silt, clay and residual bitumen. The terrestrial reclamation can begin after sufficient consolidation of fluid tailings streams. Oil sands mine operators use various technologies to facilitate dewatering and consolidation of fluid tailings (Wang et al., 2014). During tailings thickening process, fluid tailings are treated with flocculants such as polyacrylamide (PAM) which bind the smaller particles together to create thickened tailings (TT) of about 45% solids content (Wang et al., 2014; Lin et al., 2015). Once TT deposits are placed in their final landscape position and meet specified performance criteria, they can be reclaimed into terrestrial landscapes (Alberta Energy Regulator, 2020, 2022). Terrestrial landform construction on tailings (i.e., revegetation on tailings after covering with a layer of suitable substrate) using native plants is an appropriate strategy for reclaiming oil sands tailings (Lalonde et al., 2020; Samad et al., 2022). Plant growth in tailings is limited by the inherent ecotoxicity from residual hydrocarbon, salts, alkalinity, and the low levels of organic matter and nutrients leading to the necessity of covering tailings with a suitable reclamation cover material before revegetation. Peat-based reclamation material, including peat mineral mix (PMM), has been shown to improve plant growth (Lalonde et al., 2020; Degenhardt et al., 2023) and reduce stresses associated with tailings in various plant communities, including different upland and wetland species (Samad et al., 2022). Native boreal upland and wetland plant communities are considered appropriate for revegetation and reclamation operations in the oil sands mining area (Dhar et al., 2018; Lalonde et al., 2020; Samad et al., 2022).

Microbial communities play pivotal roles in soil nutrient cycling and plant growth and have been shown to shift in response to physicochemical and biological variables during the ecological restoration of disturbed sites (Wu et al., 2020; Gazitúa et al., 2021). Tailings streams with different age, processing, and management histories can host distinct microbial communities which may influence the long-term reclamation efforts (Foght et al., 2017). Previous research on the microbiology of tailings has mainly focused on the indigenous microbial communities of tailings ponds and has found that these communities can impact tailings management by affecting tailings chemistry and greenhouse gas emissions (Wilson et al., 2016; Foght et al., 2017). However, microbial responses to thickened tailings in a terrestrial reclamation setting with native boreal plant species remain largely unknown. Some soil microorganisms, including plant growth-promoting bacteria (PGPB) can promote plant growth through several mechanisms such as: fixing atmospheric nitrogen, solubilizing phosphates, secreting phytohormones (auxin, cytokinin, gibberellin), lowering plant ethylene levels by ACC deaminase production, and sequestrating iron through siderophores production (Olanrewaju et al., 2017; Poria et al., 2022). In addition to PGPB, some fungi, such as arbuscular mycorrhizal fungi (AMF) can enhance plant nutrient uptake and growth (Gamalero et al., 2004; del Barrio-Duque et al., 2019). Indeed, it has been reported that arbuscular mycorrhizal fungi can increase root branching, resulting in increased plant nutrient uptake (Berta et al., 2002; Gamalero et al., 2004). There is increasing evidence that microorganisms are critical to improving plant productivity in harsh environments (Harris, 2009; Vimal et al., 2017; Gazitúa et al., 2021) and play a central role in soil mineral nutrient availability in soils of the oil sands region (MacKenzie and Quideau, 2010). Moreover, tailings associated microorganisms can metabolize toxic and recalcitrant constituents found in oil sands tailings (Chegounian et al., 2020; Saborimanesh, 2021).

Various studies have shown that vegetation influences the development of microbial communities in disturbed sites (Dimitriu et al., 2010; Gagnon et al., 2020b). Furthermore, plants can influence plant-microbial relationship by altering the quantity and quality of organic matter entering the soil via root organic matter, exudates, and leaf litter (Hargreaves and Hofmockel, 2014). Thus, the variation in plant communities has important implications for microbial communities and nutrient cycling. Additionally, the placement of organic capping (e.g., PMM) over tailings can modulate the response of microorganisms by modifying soil properties and plant growth (Jamro et al., 2014; Lalonde et al., 2020). Microbial communities can be affected by various factors, including soil amendments such as forest floor-mineral mix (FFMM) and PMM (Ma et al., 2017; Mitter et al., 2017; Stefani et al., 2018), vegetation (Gagnon et al., 2020b), soil rehabilitation time (Birnbaum et al., 2017) and other physiochemical properties including pH, salinity, moisture, aeration, etc. (Xue et al., 2018; Gschwend et al., 2021). In fact, soil pH is considered one of the key drivers of microbial community composition (Rousk et al., 2010b). Salinity has also been demonstrated as a dominant factor affecting soil microbial populations (Rath et al., 2019; Yang and Sun, 2020). In addition, soil moisture and aeration have been reported to impact microbial community structure (Meisner et al., 2021). For instance, high soil aeration was shown to stimulate microbial biomass and shift the composition of microbial communities (Picek and Simek, 2000).

Previous studies concerning oil sands tailings reclamation primarily looked at plant community establishment and assemblage rather than microbial community dynamics (Rowland et al., 2009; Errington and Pinno, 2015; Dhar et al., 2018, 2020). However, microbial communities can contribute considerably to tailings restoration outcomes through nutrient cycling and complex mutualistic interactions with plants (Harris, 2009; Gazitúa et al., 2021). The primary goal of this study was therefore to understand the response of tailings bacterial and fungal communities to different capping and vegetation strategies over time. For this purpose, a mesoscale greenhouse experiment was sampled for two growing seasons to evaluate the response of tailings and PMM-associated microbial communities during the establishment of boreal upland and wetland species grown directly in tailings or in tailings capped with PMM. The combined knowledge of aboveground biomass and belowground soil–plant-microorganism interactions will guide the development of effective strategies to reclaim sustainable landscapes in the oil sands mining area.

## 2. Materials and methods

### 2.1. Greenhouse experiment

**Figure 1:**
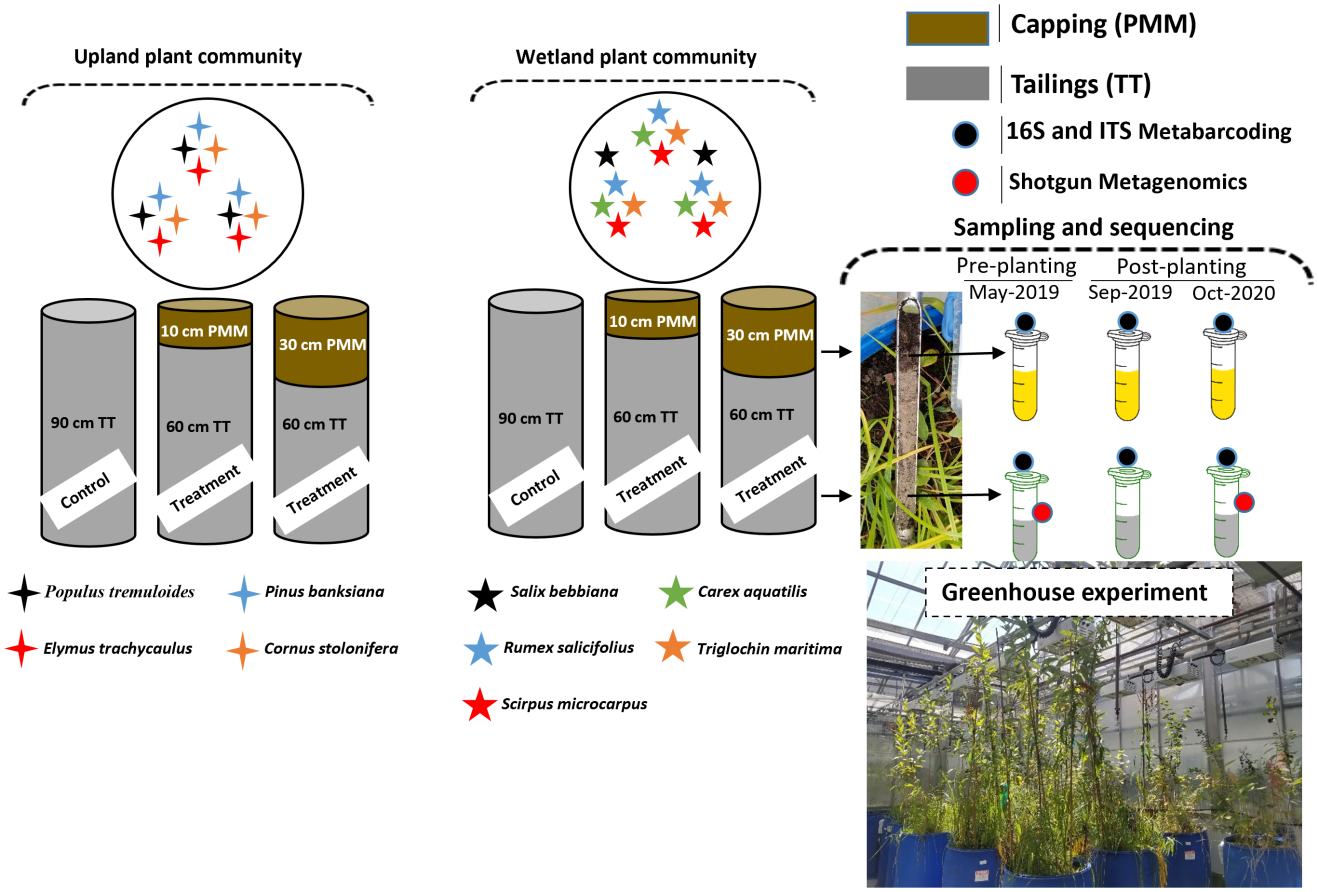
Diagram of experimental design in which upland and wetland plant communities were grown on thickened tailings with or without PMM capping of different depths (10 and 30 cm). A total of 12 plants (indicated by 4-point stars) per column belonging to four different upland plant species and 14 plants (indicated by 5-point stars) per column belonging to five different wetland plant species were planted in upland and wetland columns, respectively. Four columns were assembled for each treatment (*n* = 4). The tailings and PMM layers were sampled at the start of the experiment (May 2019; Pre-planting baseline), after the first growing season (Sep 2019; GS1), and after the second growing season (Oct 2020; GS2). All samples were subjected to 16S and ITS metabarcoding (indicated by black circles). Tailings samples from baseline and GS2 were also sequenced by shotgun metagenomics (indicated by red circles). TT, thickened tailings; PMM, peat mineral mix.

For this experiment, columns (plastic cylindrical barrels of 208 L volume, 54 cm in diameter and 90 cm in height) containing thickened tailings (TT) alone or TT capped with two different depths (10 or 30 cm) of peat mineral mix (PMM) were assembled in 2019 and planted with two distinct plant communities (upland and wetland). The upland community was composed of four different upland plant species (a total of 12 plants per column) including *Populus tremuloides* Michx (aspen), *Elymus trachycaulus* ssp. *trachycaulus* (Link) Malte (slender wheat grass), *Pinus banksiana* Lamb (jack pine), and *Cornus sericea* L. also referred to as *Cornus stolonifera* Michx. (red-osier dogwood). The wetland community was composed of five different plant species (a total of 14 plants per column) including *Salix bebbiana* Sarg. (Bebb’s willow), *Rumex salicifolius* Weinm. (willow dock), *Scirpus microcarpus* J. Presl and C. Presl (Small-fruited bulrush), *Carex aquatilis* Wahlenb (water sedge) and *Triglochin maritima* L. (sea-side arrow grass). Four replicate columns were assembled for each combination of plant community and capping treatment, for a total of 24 columns. More details about the treatments can be found in Figure 1. TT and capping material (PMM) were received from Imperial Oil Ltd., Calgary, Canada. TT has a loamy sand texture with a sand to fines ratio (SFR) of 2.8. The basic physicochemical properties of tailings and PMM are described in Table 1. The columns were placed in a greenhouse set at a temperature of 19–21°C with 65–75% humidity and a photoperiod of 12 h. Overhead irrigation using reverse osmosis water was applied consistently to all columns to mimic precipitation, and the only loss of water was through evapotranspiration since the columns were an enclosed system with no drainage. Moreover, moisture conditions in the top 20 cm for each column were assessed weekly using a TDR 100 Soil Moisture Tester (Spectrum Technologies, Inc., United States); these values were used to inform if additional hand watering was required to maintain a range of volumetric water contents (VWCs) between 19–24% and 25–30% for the upland and the wetland columns, respectively. No fertilization was applied. Soil temperature logging sensors were installed during column setup in three of the total four replicates for each treatment. The EM50 loggers recorded hourly moisture and temperature data from three 5TM sensors installed at 20, 40, and 60 cm below the surface (METER Environment, United States), and average temperature (Supplementary Figure S1) and average moisture (Supplementary Figure S2) were recorded at each depth. Plant growth and survival were recorded before sampling (Supplementary Figure S3). The tailings and PMM (capping) were sampled (*n* = 4) using a sterile core sampler at the start of the experiment pre-planting (May 2019; Baseline), after the first growing season (Sep 2019; 5 months after plantation; GS1), and after the second growing season (Oct 2020; 18 months after plantation; GS2). The samples were collected using a small soil corer, transferred to a 50 mL Falcon tube, immediately placed on ice, and stored at −20°C until further processing.

**Table 1:** Basic properties of thickened tailings (TT) and peat mineral mix (PMM); mean ± one standard deviation of the mean.

Main properties	Parameter	PMM	TT
Physicochemical	pH	6.9 ± 0.2	8.7 ± 0.1
	Electrical conductivity (dS/m)	0.5 ± 0.1	1.0 ± 0.1
	Sodium adsorption ratio	0.2 ± 0.02	1.9 ± 0.1
	Cation exchange capacity (cmol/kg)	43.8 ± 10.2	3.0 ± 1.1
	Solids (%)		79.3 ± 1.0
	Sand to fines ratio		2.8 ± 0.5
	% sand (2 mm – 50 μm)		70 ± 7
	% silt (2–50 μm)		17 ± 3
	% clay (<2 μm)		14 ± 5
Inorganics	Exchangeable Ca^2+^ (mg/kg)	8,820 ± 1,148	332.8 ± 52.2
	Exchangeable Na^+^ (mg/kg)	87.9 ± 10.2	156.8 ± 17.1
	Exchangeable K^+^ (mg/kg)	67.0 ± 13.8	47.5 ± 6.2
	Exchangeable Mg^2+^ (mg/kg)	1,261 ± 23	106.2 ± 17.4
	Extractable P (mg/kg)	2.1 ± 0.3	2.3 ± 0.2
	Extractable S (mg/kg)	43.7 ± 10.7	71.8 ± 7.4
	NO_3_-N	22.1 ± 9.1	3.0 ± 1.0
	NH_4_-N		10 ± 1
	Total organic matter	16.6 ± 2.8	1.4 ± 0.3
	Total carbon	8.6 ± 1.4	0.9 ± 0.3
	Total nitrogen	0.3 ± 0.04	0.005 ± 0.005
Organics	PHC F1 C6-C10 (mg/kg)		<DL
	PHC F2 C10-C16 (mg/kg)		521 ± 197
	PHC F3 C16-C34 (mg/kg)		3,689 ± 1,316
	PHC F4 C34-C50+ (mg/kg)		1862 ± 673
	PHC F5 (mg/kg)		1816 ± 670
	Naphthenic acids (mg/kg)		227 ± 85
	Bitumen (%)		0.61 ± 0.2
Trace metals	Boron (mg/kg)		1.0 ± 0.2
	Barium (mg/kg)		0.1 ± 0.01
	Copper (mg/kg)		0.02 ± 0.003
	Iron (mg/kg)		0.02 ± 0.009
	Manganese (mg/kg)		1.2 ± 0.7
	Strontium (mg/kg)		1.1 ± 0.2
	Zinc (mg/kg)		<DL

Ca^2+^, calcium ion; Na^+^, sodium ion; K^+^, potassium ion; Mg^2+^, magnesium ion; P, phosphorous; S, sulfur; NO_3_-N, nitrate nitrogen; NH_4_-N, ammonium nitrogen; PHC, petroleum hydrocarbons.

### 2.2. DNA extraction and metabarcoding

A metabarcoding approach was used to characterize the microbial communities in TT and in PMM capping material. DNA was extracted from the samples (0.25 g for TT and 0.1 g for PMM) using the DNeasy PowerSoil kit on a QIAcube automated platform (Qiagen, Toronto, ON) following the manufacturer’s instructions. DNA from each sample was quantified with the Qubit dsDNA HS (for tailings) or BR (for PMM) assay kit (Thermo Fisher Scientific, United States) using a Qubit Fluorometer 2.0 (Life Technologies, Burlington, ON, Canada). Primers 515F-Y (5′-GTGYCAGCMGCCGCGGTAA-3′) and 926R (5′-CCGYCAATTYMTTTRAGTTT-3′) targeting the V4-V5 regions of the 16S ribosomal RNA gene of bacteria and archaea (Parada et al., 2016) and primer pairs ITS9 (5′-GAACGCAGCRAAIIGYGA-3′) (Menkis et al., 2012) and ITS4 (5′-TCCTCCGCTTATTGATATGC-3′) targeting the ITS2 region of fungi (White et al., 1990; Rivers, 2016) were used in this study. Library preparation for Illumina sequencing was performed using the above-mentioned primers according to the manufacturer’s instructions (Illumina, 2013). The reactions for amplicon PCR were set up by mixing 25 μL of HotStarTaq Plus Master Mix (QIAGEN, Inc., Germantown, MD, United States), 19 μL RNase-free water, 0.5 μL of each 10 μM primer and 5 μL of gDNA normalized at 5 ng μL^−1^ Thermal cycler (C1000 Touch Thermal Cycler, BIO-RAD) conditions were as follows: 95°C for 5 min; 95°C for 45 s; 50°C for 45 s (16S primers, 35 Cycles and ITS2 primers, 40 cycles); 72°C for 1 min; and final elongation at 72°C for 10 min. PCR products were purified using magnetic beads solution (81 μL/sample; Agencourt AMPure XP), then unique indexes were added to each sample using the Nextera XT Index Kit v2, in accordance with Illumina’s protocol (Illumina, 2013). After index PCR, the amplicons were purified again with magnetic beads (56 μL/sample), quantified using the Synergy™ Mx Microplate Reader (BioTek Instruments, Inc., Winooski, VT, United States), and combined at equimolar concentration. Paired-end sequencing (2 × 300 bp) was performed on an Illumina MiSeq platform at the Next Generation Sequencing Platform of the CHU de Québec-Université Laval Research Centre. Sequences were deposited in the NCBI Sequence Read Archive under BioProject number PRJNA894999 (accession number SAMN31487590 to SAMN31487742).

### 2.3. Shotgun metagenomic sequencing

A metagenomics approach was applied to a subset of tailings samples to investigate the treatment effects on microbial functions. Shotgun metagenomic libraries were prepared using the Nextera XT DNA library preparation kit and the Nextera XT Index kit v2 (Illumina) following the manufacturer’s instructions. The resulting DNA libraries were purified with Agencourt AMPure XP beads (Beckman Coulter, Inc., United States) and fragment size distribution within the range of 250–1,000 bp was verified by running the libraries on the 2,100 Bioanalyzer using the Agilent high sensitivity DNA kit. Libraries were quantified with the Qubit BR dsDNA assay kit and manually normalized to ensure equal library representation in the pool prior to sequencing. Normalized libraries were sequenced on an Illumina NovaSeq 6,000 using a S4 PE 2 × 150 bp flow cell at the Centre d’Expertise et de Services Génome Québec. Sequences were deposited in the NCBI Sequence Read Archive under BioProject number PRJNA894999 (accession number SAMN31487743 to SAMN31487757).

### 2.4. Bioinformatics and statistical analysis of metabarcoding data

Analysis of metabarcoding sequences was performed with QIIME 2 (Bolyen et al., 2019). Raw sequences were imported with the “Casava 1.8 paired-end demultiplexed fastq” function, and the files were stored into Qiime2 artifacts (demux.qza). Data were demultiplexed and quality filtered using the q2-demux plugin, followed by denoising with DADA2 using the “qiime dada2 denoise-paired” function (Callahan et al., 2016). Taxonomy was assigned to ASVs using the naïve Bayes classifier (q2-feature-classifier) (Bokulich et al., 2018) using SILVA 138 (Yilmaz et al., 2014) and UNITE 8.2 (Abarenkov et al., 2010) databases for 16S and ITS, respectively. The classifier Qiime2-compatible database files were obtained from Qiime2 data resources.^1^ Then taxonomy-based filtering of the feature table and feature data was performed to remove sequences and counts corresponding to non-target amplification such as chloroplasts, mitochondria, and sequences unidentified at the kingdom level. About one-third of ITS reads in tailings samples were assigned to ciliated protists Ciliophora and were removed from the ITS dataset before further analysis. The “qiime tools export” function was used to export ASV tables generated by QIIME2, which were further imported into R version 4.2.1 (R Core Team, 2022) for taxonomic composition, alpha diversity and beta diversity analysis. The R package phyloseq and qiime2R were used for data handling (McMurdie and Holmes, 2013; Bisanz, 2022), and all plots were created using the ggplot2 package (Wickham, 2016).

To analyze alpha diversity (Observed ASVs, Shannon index and Pielou’s evenness), datasets were rarefied to the minimum sample size, 6,402 ASVs per sample for bacteria and 2,527 ASVs per sample for fungi (Weiss et al., 2017). Prior to statistical analyses, parametric assumptions of data normality were verified by Shapiro–Wilk test of normality using the “shapiro.test” function. Homoscedasticity was checked using the Bartlett test (“bartlett.test” function). Akaike’s Information Criterion (AIC) was used for the model selection and the model with lowest AIC value was used for forecasting. Generalized least squares (gls) linear models (package nlme) were fitted, with weights allowing different variances for treatment in case of heteroscedasticity. Three-way ANOVA (function: anova, package: stats) was applied to determine how capping treatments (0, 10, 30 cm), plant communities (upland, wetland), post-planting time points (Sep 2019, Oct 2020) and their interaction influence alpha diversity indices. When the main effects were significant but interaction effects were not, multiple comparisons of the means were done on the main effects only, while in the presence of significant interaction effects, multiple comparisons were done within factors involved in the significant interactions. Additionally, one-way ANOVAs were applied on three sampling time points including pre-planting baseline samples (May 2019), GS1 (Sep 2019), and GS2 (Oct 2020) to test how alpha diversity changes after planting when compared to baseline samples. The gls models were fitted (nlme package), taking temporal autocorrelations into account using corCAR1 function (Pinheiro et al., 2022). The R package emmeans was used for *post hoc* multiple comparisons among groups and FDR-adjusted *p* < 0.05 were considered statistically significant. For beta diversity analysis, ASV tables (non-rarefied) were normalized using the geometric mean of pairwise ratios (GMPR) method (Chen et al., 2018). Beta-diversity was calculated as the Bray–Curtis dissimilarity index (function: distance, package: phyloseq) and visualized using a non-metric multidimensional scaling plot (NMDS, “metaMDS” function, “vegan” package). Permutational Multivariate Analysis of Variance was performed using Bray–Curtis dissimilarity matrices with 9,999 permutations (PERMANOVA function: adonis, package: vegan). Comparisons between groups were checked by a multilevel pairwise comparison test (“pairwise.adonis2” function, “pairwiseAdonis” package). Variance heterogeneity between groups was tested with the function “betadisper” in the “vegan” package. To identify the taxa whose relative abundances differed between the capping treatments (10 and 30 cm) and control (0 cm), differential abundance analysis (DAA) was conducted using ANCOM-BC (Analysis of compositions of microbiomes with bias correction) package in R (Lin and Peddada, 2020). Rare taxa with less than 10 counts were removed, and taxa were considered as differentially abundant only if Benjamini-Hochberg adjusted *p*-values were significant (*p* < 0.05). A total of 80 samples, 48 from the TT layer and 32 samples from the PMM layer, were sequenced. Six samples of the PMM layer from GS1 sampling were removed due to low read abundance.

### 2.5. Shotgun metagenomic data analysis

Metagenomics data were processed through a previously described bioinformatic pipeline (Tremblay et al., 2017). Briefly, Sequencing adapters in all reads were removed, and bases with low-quality scores (<30) at the end of reads were cut using Trimmomatic 0.39 (Bolger et al., 2014) to generate quality-controlled (QC) reads. QC-passed reads from each sample were co-assembled using Megahit 1.2.9. Genes were predicted from contigs, and good quality reads used for co-assembly input were mapped back onto contigs to evaluate contig and gene abundances. The counts of QC-passed reads are summarized in Supplementary Table S1 for all metagenomic sequencing libraries. Gene prediction on the obtained contigs was performed by calling genes on each assembled contig using Prodigal v2.6.3 (Hyatt et al., 2010). Genes were annotated following the JGI’s guidelines (Huntemann et al., 2016) using six different databases: (1) RPSBLAST (v2.2.29+; Camacho et al., 2009) against COG database (v3.11); (2) RPSBLAST (v2.2.29+) against KOG database (v3.11); (3) HMMSCAN (v3.1b1; Eddy, 2011) against PFAM-A v27.0 database (Finn et al., 2014); (4) TIGRFAM database v15.0; (5) BLASTP (v2.2.29+) against KEGG database (v71.0) and (6) BLASTN (v2.2.29+) against NCBI’s nucleotide (nt) database (Li and Durbin, 2010). For each database comparison, the best hit having at least an e-value ≥0.01 was kept for each query. Alignment files in bam format were sorted by reads coordinates using SAMtools v1.1,^2^ and only properly aligned read pairs were kept for downstream steps (Supplementary Table S1). Each bam file (containing properly aligned paired-reads only) was analyzed for coverage of called genes and contigs by bedtools 2.23 (Quinlan and Hall, 2010) using a custom bed file representing gene coordinates on each contig. Only paired reads overlapping their contigs or genes were considered for gene counts.

Contigs (not genes) sequences were also blasted against NCBI’s nt database for taxonomic assignment. Taxonomy of each contig was assigned using the NCBI taxonomy database (Sayers et al., 2011).^3^ GenInfo Identifier (GIs) resulting from BLASTN against nt was used to retrieve full taxonomic lineage (when available) from the NCBI taxonomy database. Taxonomic lineages were integrated into the contig abundance of read counts matrix to generate an OTU table format file (with contigs replacing OTUs as rows). Taxonomic summaries were performed using a combination of in-house Perl, R scripts, and Qiime v.1.9.0 (Caporaso et al., 2010). The R software package Deseq2 (Love et al., 2014) was used for differential abundance analysis of genes (log fold-change = 2, False Discovery Rate < 0.05) to identify significantly enriched or depleted potential genes and pathways in different capping treatments and control comparisons. Differentially abundant genes were grouped according to KEGG database functional categories (Kanehisa, 2000).

## 3. Results

### 3.1. Microbial diversity in the tailings

**Figure 2:**
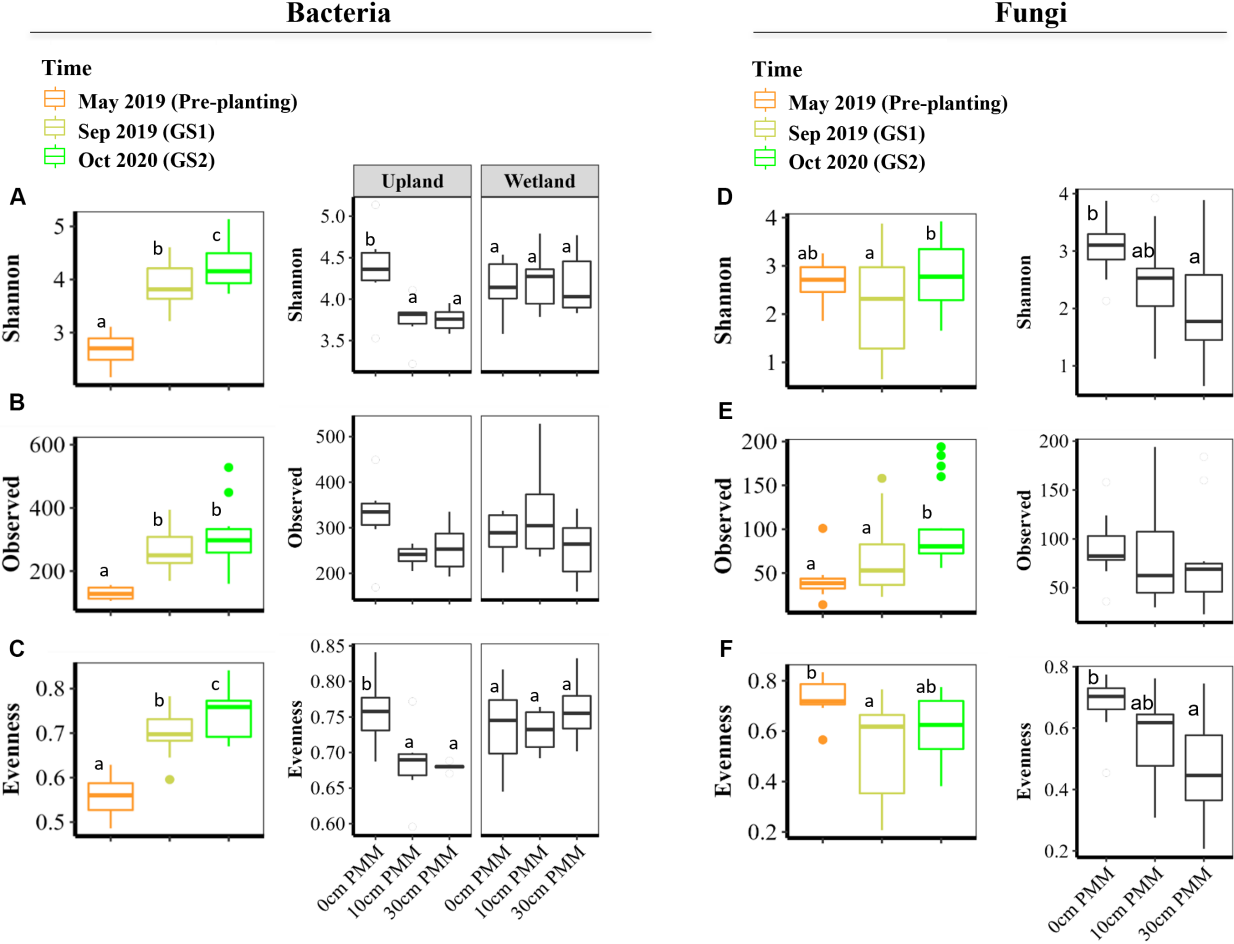
Alpha diversity indices of bacterial and fungal communities in thickened tailings (TT) samples, determined by Observed ASVs (i.e., richness), Shannon diversity, and Pielou’s evenness for bacteria **(A–C)** and fungi **(D–F)**. Generalized least squares (gls) models and three-way ANOVA was applied (see Supplementary Table S2) to determine how capping treatments (0, 10, 30 cm), plant communities (upland, wetland) and time points (Sep 2019, Oct 2020) and their interaction influence alpha diversity indices (*n* = 3). One-way ANOVA was applied to test how microbial communities changed after each growing season compared to pre-planting baseline samples (May 2019). FDR, false discovery rate; May 2019, baseline or pre-planting; Sep 2019, GS1; Oct 2020, GS2.

Bacterial and fungal alpha diversity was measured by Observed ASVs (richness), Shannon’s diversity index (Shannon), and Pielou’s evenness index (evenness). Time was identified as the most important driver of bacterial alpha diversity (Figure 2), with overall lower bacterial alpha diversity recorded in the tailings at the beginning of the experiment (May 2019; pre-planting baseline samples). Bacterial richness, Shannon diversity and evenness increased significantly after the first growing season (Sep 2019; GS1) when compared to baseline samples (Figure 2). Moreover, Shannon and evenness significantly increased between GS1 and GS2.

The main effects of capping treatments on bacterial alpha diversity were not significant. However, a significant interaction effect between plant community and capping treatment was detected for Shannon and evenness (Figure 2 and Supplementary Table S2). Further testing of the impact of capping treatment within each plant community type showed a negative impact of capping on bacterial alpha diversity of the tailings in the presence of the upland plant community, while no significant effect of capping was detected for the wetland plant community. The plant community itself (upland vs. wetland) only affected the evenness but did not affect the richness and Shannon (Supplementary Table S2). Capping depth (10 cm vs. 30 cm) had no significant effect on bacterial alpha diversity (Figures 2A–C).

Fungal alpha diversity showed a slightly different response than bacterial alpha diversity (Figures 2D–F and Supplementary Table S2). While the fungal richness and Shannon diversity index significantly increased between GS1 and GS2 (post-planting), fungal evenness was not significantly changed (Figures 2D–F). Unlike bacterial evenness, fungal evenness significantly decreased after GS1 compared to pre-planting baseline samples. Fungal richness was not significantly affected by capping treatment nor by plant community. On the other hand, both Shannon and evenness were significantly affected by capping treatment, with lower values detected in columns with capping compared to the controls with no cap. Capping depth as well as plant community had no significant effect on fungal alpha diversity (Figures 2D–F and Supplementary Table S2).

**Figure 3:**
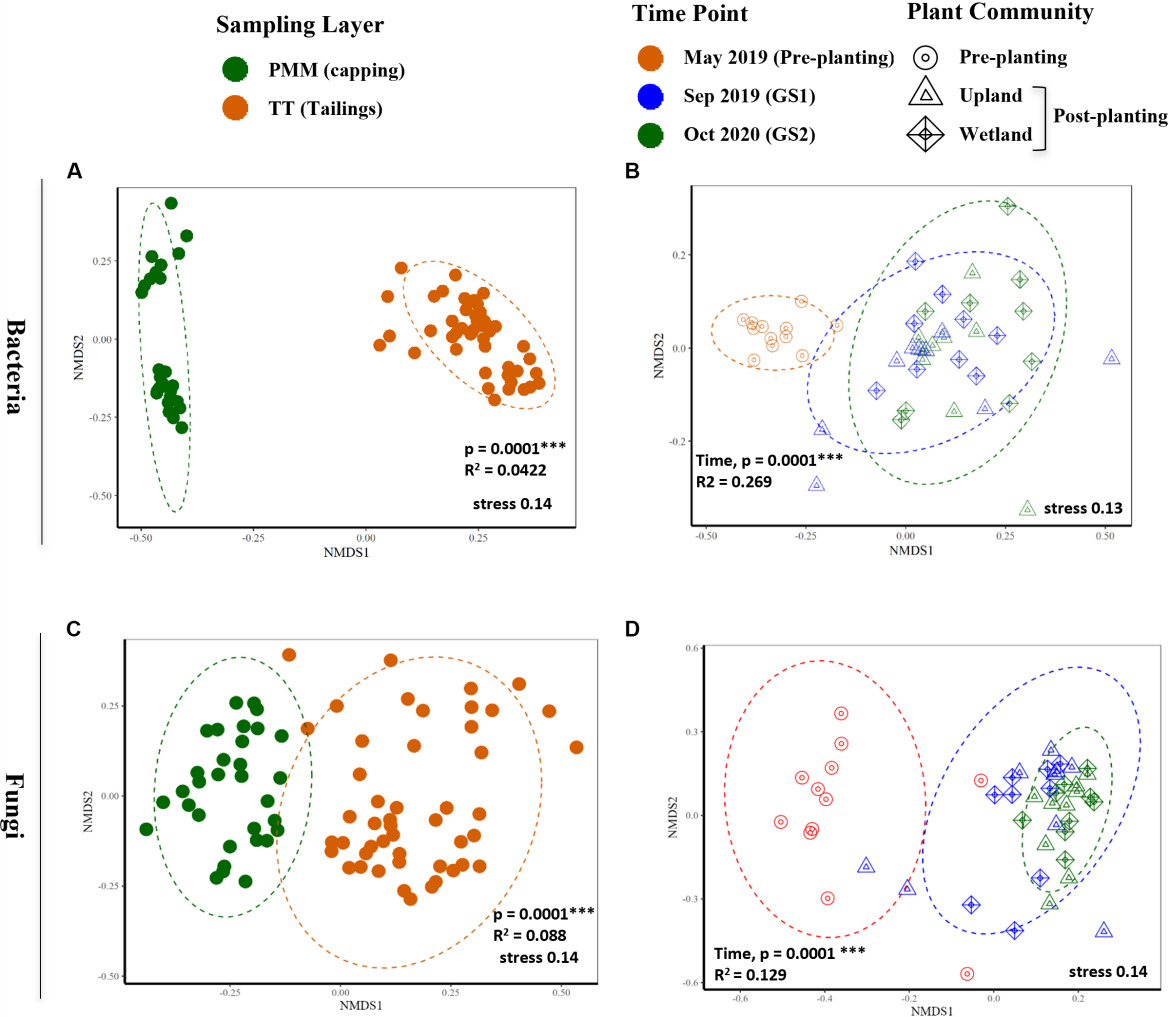
Non-metric multidimensional scaling (NMDS) based on Bray-Curtis dissimilarities of bacterial **(A,B)** and fungal **(C,D)** communities. **(A,C)** Differences between capping (PMM) and thickened tailings (TT) samples for bacterial and fungal communities, respectively. **(B,D)** Effect of time on TT samples for bacterial and fungal communities, respectively. *p-*values indicate statistical differences between capping layers **(A,C)** and time points **(B,D)** according to PERMANOVA. FDR-adjusted *p* < 0.05 was considered statistically significant, as indicated by asterisk, * < 0.05, ** < 0.005, *** < 0.0005. FDR, false discovery rate. Detailed results of PERMANOVA are provided in Table 2.

Similarly, beta diversity after planting was significantly changed compared to pre-planting baseline for both bacterial and fungal communities (Figure 3 and Table 2). GS1 and GS2 samples were significantly different compared to the baseline samples for both groups, but the difference between GS1 and GS2 samples was significant only for bacteria in wetland columns (Table 2 and Figure 3). While the main effects of the capping treatments on bacterial and fungal beta diversity were not significant, the main effects of plant community and the interaction effects between capping treatment and plant community were significant (Table 2). Further pairwise comparisons within each plant community showed that capping treatment had a significant effect on bacterial and fungal beta diversity. The effect of capping depth (10 cm vs. 30 cm) on beta diversity was only observed in upland columns.

**Table 2:** Results of permutational multivariate analysis of variance (PERMANOVA, 9999 permutations) on Bray–Curtis dissimilarity of bacterial and fungal communities as a function of capping treatments (0, 10, 30 cm), plant communities (upland, wetland), post-planting time points (Sep 2019, Oct 2020), and their interactions (*n* ≥ 3).

Pre-planting vs. Post-planting	16S-Bacteria				ITS2-Fungi			Df	SS	*F*	*p*	SS	*F*	*p*
Time	2	2.488	9.711	**1.00E-04**	1.966	0.104	**0.0001**
Contrasts *a priori*							
May 2019 vs. Sep 2019 (GS1)	1	1.556	0.277	**0.001**	1.296	0.104	**0.001**
May 2019 vs. Oct 2020 (GS2)	1	2.110	0.349	**0.001**	1.386	0.117	**0.001**
Post-planting							
Time	1	0.239	1.756	**0.0286**	0.357	0.104	0.1218
Capping	2	0.384	1.410	0.0568	0.657	1.223	0.1486
Plant community	1	0.237	1.741	**0.0297**	1.009	3.756	**0.0001**
Capping × Plant community	2	0.447	1.642	**0.0126**	1.470	2.737	**0.0002**
Contrasts *a priori*							
Upland: 0 cm vs. 10 cm	1	0.400	0.186	**0.0104**	0.872	0.209	**0.0024**
Upland: 0 cm vs. 30 cm	1	0.430	0.232	**0.0019**	1.275	0.331	**0.0014**
Upland: 10 cm vs. 30 cm	1	0.192	0.205	**0.0024**	0.461	0.196	**0.0017**
Wetland: 0 cm vs. 10 cm	1	0.338	0.202	**0.0021**	0.876	0.259	**0.0023**
Wetland: 0 cm vs. 30 cm	1	0.312	0.176	**0.0024**	1.007	0.280	**0.0031**
Wetland: 10 cm vs. 30 cm	1	0.179	0.105	0.2604	0.247	0.088	0.4321
Plant community × Time points	1	0.388	2.854	**0.0005**	0.393	1.463	0.0866
Contrasts *a priori*							
Upland: Sep 2019 vs. Oct 2020	1	0.189	1.216	0.2397	–	–	–
Wetland: Sep 2019 vs. Oct 2020	1	0.306	2.073	**0.0079**	–	–	–

FDR-adjusted *p*-value < 0.05 was considered statistically significant. Only significant interaction effects are reported. May 2019, pre-planting baseline; Sep 2019, GS1; Oct 2020, GS2; SS, sum of squares; F, pseudo-*F-*value; *p* = FDR corrected *p*-value; FDR, false discovery rate. Values in bold indicate statistical significance with FDR corrected *p*-value < 0.05.

### 3.2. Microbial diversity in the capping material

Bacterial and fungal alpha diversity (Observed ASVs and Shannon) was significantly higher in capping material (PMM) than in tailings (Supplementary Figure S4). Alpha diversity was not significantly impacted by time in the capping material and remained relatively stable temporally when compared to the tailings layer (Supplementary Figure S4). Likewise, the beta diversity of the capping material was also significantly different from the tailings layer for both bacteria and fungi, with capping samples showing a clear separation from the tailings samples in ordinations (Figures 3A,B). However, time (Sep 2019 and Oct 2020), capping depth (10 and 30 cm) and plant communities (upland and wetland) had no impact on the beta diversity of capping material (Supplementary Table S3).

### 3.3. Microbial taxonomic profiles

**Figure 4:**
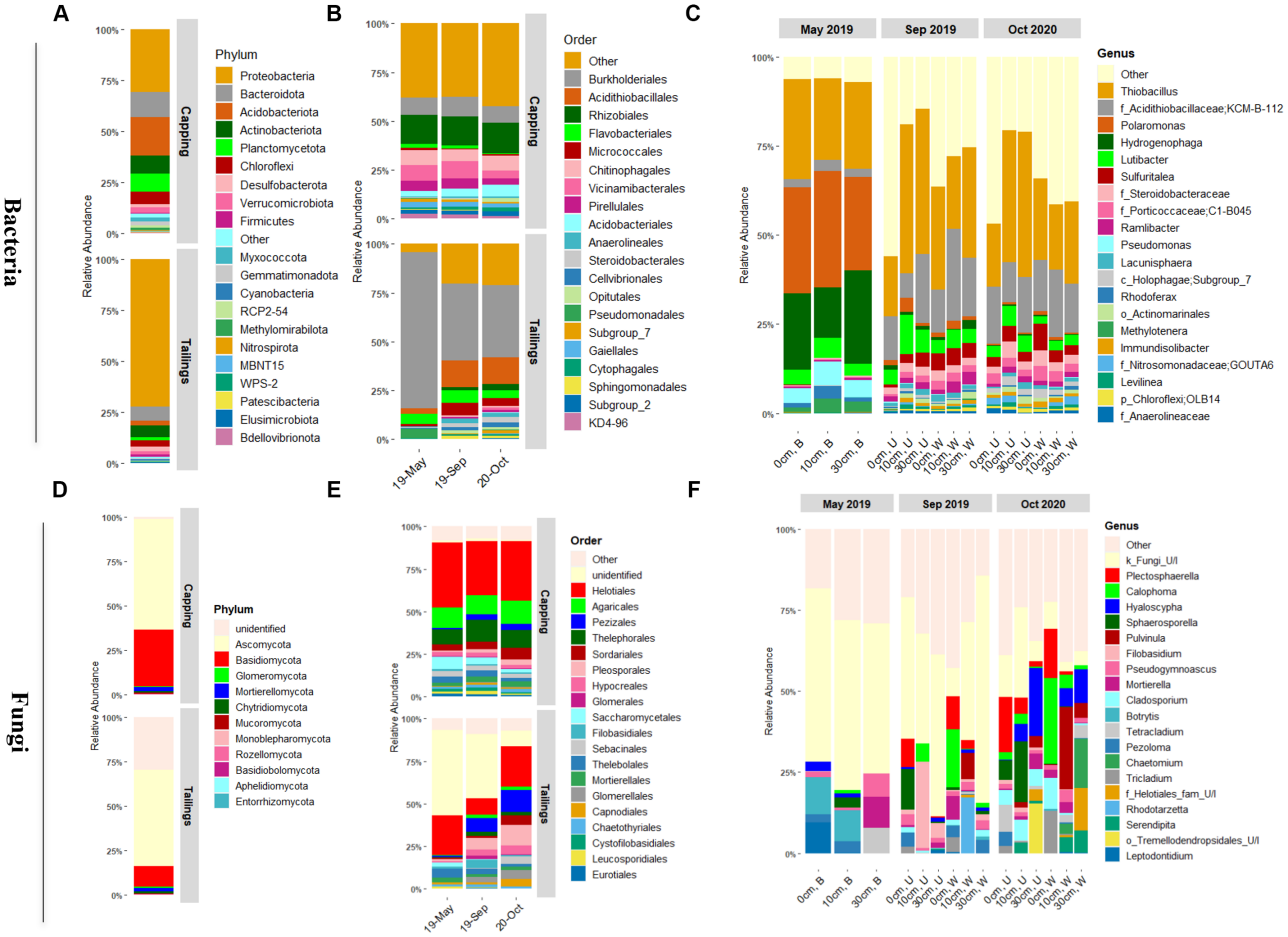
Taxonomic profiles of microbial communities in thickened tailings and capping material (PMM) as assessed by sequencing the 16S rRNA gene for bacteria **(A–C)**, and ITS2 region for fungi **(D–F)**. **(A,D)**, taxonomic profiles at the phylum level across all samples for the capping (PMM) and tickened tailings (TT) layers. **(B,E)**, taxonomic profiles at the order level as a function of time for the capping and tailing layers. **(C,F)**, taxonomic profiles at the genus level as a function of time, vegetation type, and capping treatment for the tailings layer only. Only 20 most abundant classified bacterial and fungal taxa are shown (*n* ≥ 3). B, baseline; W, wetland; U, upland; 0 cm, control; U/I, unidentified.

**Figure 5:**
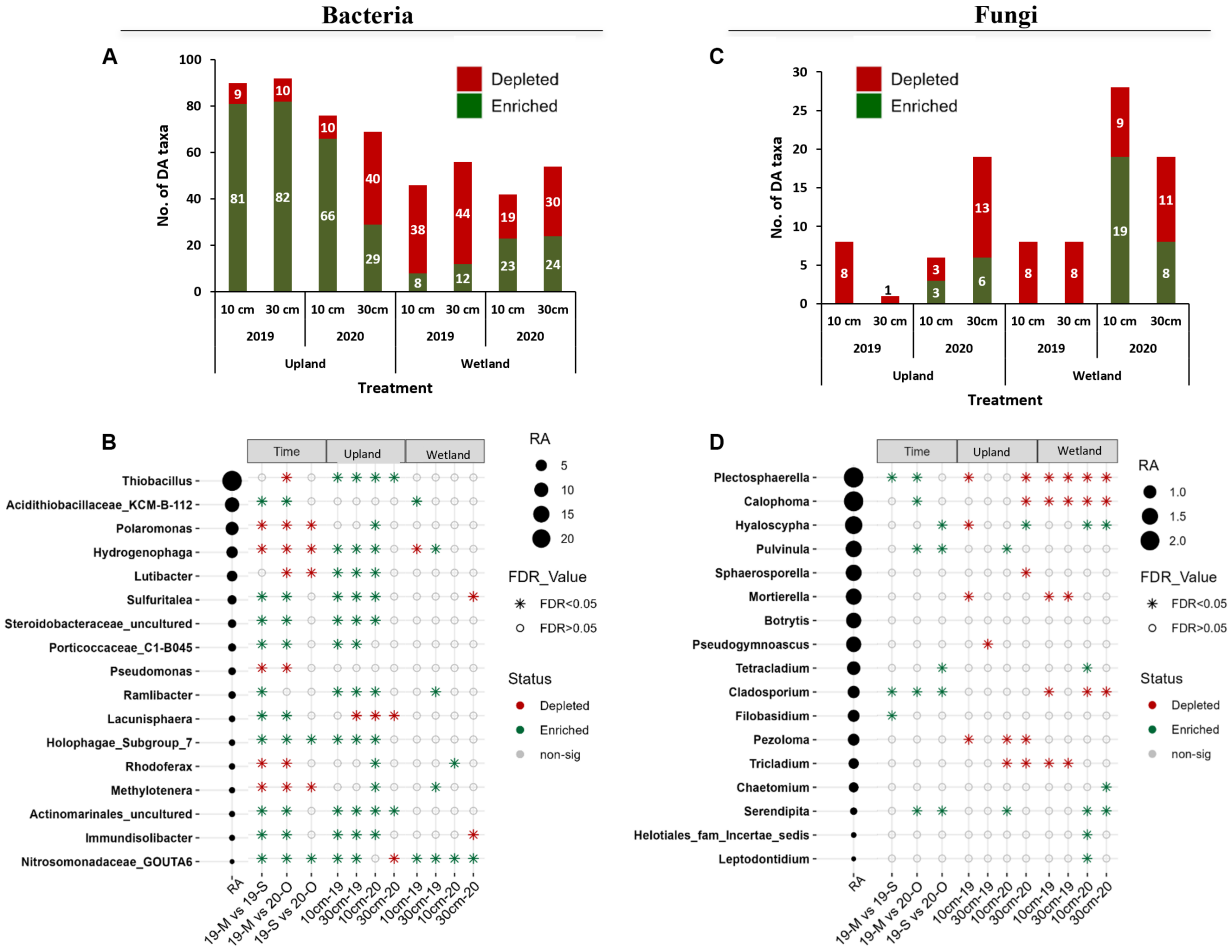
Differential abundance analysis as determined by the ANCOM-BC method for bacteria **(A,B)** and fungi **(C,D)**. **(A,C)** Total number of significantly enriched or depleted genera for capping treatments (10 cm vs. control and 30 cm vs. control). **(B,D)** List of significantly enriched or depleted genera for time point (May 2019 vs. Sep 2019, May 2019 vs. Oct 2020, and Sep 2019 vs. Oct 2020) and capping treatments (10 cm vs. control and 30 cm vs. control). Taxa with more than 0.05% relative abundance (RA) are sorted with decreasing RA. Taxa were considered as differentially abundant only if FDR adjusted *p*-values were significant (*p* < 0.05). 19-M, May 2019 (baseline); 19-S, Sep 2019 (GS1); 20-O, Oct 2020 (GS2); 0 cm, control; DA, differentially abundant; FDR, false discovery rate; AN-COMBC, analysis of the composition of microbiomes with bias correction.

Bacterial taxonomic profiles in capping material and tailings were clearly distinct but dominated mainly by four bacterial phyla: the Proteobacteria, Bacteroidota, Acidobacteriota, and Actinobacteria (Figure 4A and Supplementary Figure S5). At a lower taxonomic level, the order *Burkholderiales* (48%; mainly *Hydrogenophilaceae* and *Comamonadaceae* families) was predominant in the tailings layer, while *Rhizobiales* (14%) was dominant in the capping layer (Figure 4B). In line with results obtained for alpha and beta diversity, significant variations in relative abundances were observed over time for bacterial communities in the tailings layer but not in the capping layer (Figure 4B and Supplementary Figure S5). In the tailings layer, the order *Burkholderiales* (particularly family *Comamonadaceae*) was predominant in the baseline samples, but its relative abundance decreased after GS1. In contrast, the relative abundances of *Acidithiobacillales*, *Rhizobiales*, and *Micrococcales* (f_*Microbacteriaceae*) increased with time compared to baseline samples (Figure 4B). Taxonomic profiles at the genus level (Figure 4C), and further differential abundance (DA) analysis (Figure 5) showed that the relative abundance of genera *Hydrogenophaga* and *Polaromonas* (family *Comamonadaceae*) significantly decreased with time. Other taxa related to sulfur oxidation, such as the *Acidithiobacillaceae* KCM-B-112 and *Sulfuritalea*, were strongly enriched after GS1 and GS2 compared to baseline tailings (Figure 5). Moreover, the relative abundance of several dominant taxa (at the genus level) such as the uncultured *Holophagae* Subgroup 7 of the Acidobacteriota and *Nitrosomonadaceae* GOUTA6 was significantly increased in tailings layer after planting (GS1 and GS2) compared to baseline samples (Figure 5). Post-planting, the relative abundance of dominant taxa was not significantly changed between GS1 and GS2, apart from *Holophagae* and *Nitrosomonadaceae* which significantly increased over time (Figure 5).

The capping treatment affected the relative abundance of dominant bacterial taxa differently depending on the plant community (Figures 4C, 5A). Indeed, the number of taxa significantly affected by the capping treatment (i.e., number of DA taxa) was much higher in the upland plant community than in the wetland plant community (Figure 5A). For example, while the relative abundance of *Thiobacillus* and *Lutibacter* was significantly higher in 10 and 30 cm capping treatments than in control (0 cm) in the presence of the upland community, no effect of capping on these taxa was detected in the presence of the wetland community (Figure 5B). In fact, in the wetland community, *Nitrosomonadaceae*_ GOUTA6 was the only taxon to be consistently affected by capping, with higher relative abundance in both capped treatments (10 and 30 cm) compared to uncapped controls in both GS1 and GS2. It is noteworthy that several of the taxa that were significantly impacted by capping treatments are involved in sulfur and nitrogen cycling, including sulfur-oxidizers (e.g., *Thiobacillus* and *Sulfuritalea* that can oxidize reduced inorganic sulfur compounds such as thiosulfate and sulfide into sulfate), ammonia-oxidizer (*Nitrosomonadaceae_*GOUTA6) and nitrogen-reducer (*Lutibacter*) (Figure 5B). Like beta diversity, the influence of capping depth on relative abundance (RA) was mainly observed in the upland columns. For example, in GS2, the RA of *Polaromonas*, *Hydrogenophaga* and *Lutibacter* was significantly higher in the 10 cm PMM capping but not in the 30 cm PMM capping treatment compared to the uncapped control (Figure 5B). Overall, the RA of individual bacterial taxa changed over time and responded to capping treatments depending upon the plant community, growing season and, to some extent, the capping depth. *Nitrosomonadaceae* was the only taxa which significantly increased with both time and capping.

Ascomycota and Basidiomycota were the predominant fungal phyla detected in both capping material and tailings (Figure 4D). In tailings however, a total of 28% of the sequences could not be identified at the phylum level, indicating the presence of a high proportion of uncharacterized fungi in this sample type. The Ascomycota relative abundance increased over time, especially in the tailings layer (Supplementary Figure S6), while the proportion of unidentified fungi decreased over time (Figure 4E and Supplementary Figure S6). At the order level, the *Helotiales* remained dominant at all time point in tailings, while the relative abundance of the *Pleosporales* and *Pezizales* increased with time (Figure 4E). In the PMM layer, the order *Helotiales* was also dominant, followed by the *Agaricales* and the *Thelephorales*, with limited changes over time (Figure 4E). Many fungal ASVs from tailings were not identified at the genus level, especially in baseline and GS1 samples. Fungal taxonomic profiles were also much more variable between samples than bacterial profiles. Overall, *Plectosphaerella* and *Calophoma* were the most dominant fungal genera in the tailings layer (Figure 4F and Supplementary Figure S6). In the capping layer, *Hyaloscypha* and *Inocybe* were the most prevalent fungal genera (Supplementary Figure S6).

Differential abundance (DA) analysis showed that a much lower number of fungal taxa than of bacterial taxa were impacted by time and by the capping treatments (Figure 5C). Like bacterial taxa, fungal taxa responded to capping mainly according to the plant community, but in this case the number of DA taxa was higher in wetland than in upland communities (Figure 5C). *Plectosphaerella* and *Calophoma* were enriched compared to baseline samples, but they were generally depleted by capping (Figures 4F, 5D). The abundance of taxa known to form close associations with plant roots, including *Hyaloscypha* and *Serendipita*, generally increased with capping and time (Figure 5D). The RA of some abundant fungal taxa, such as *Plectosphaerella* and *Calophoma* was significantly decreased by capping, mainly in the wetland treatments (Figure 5D).

### 3.4. Shotgun metagenomic profiles

**Figure 6:**
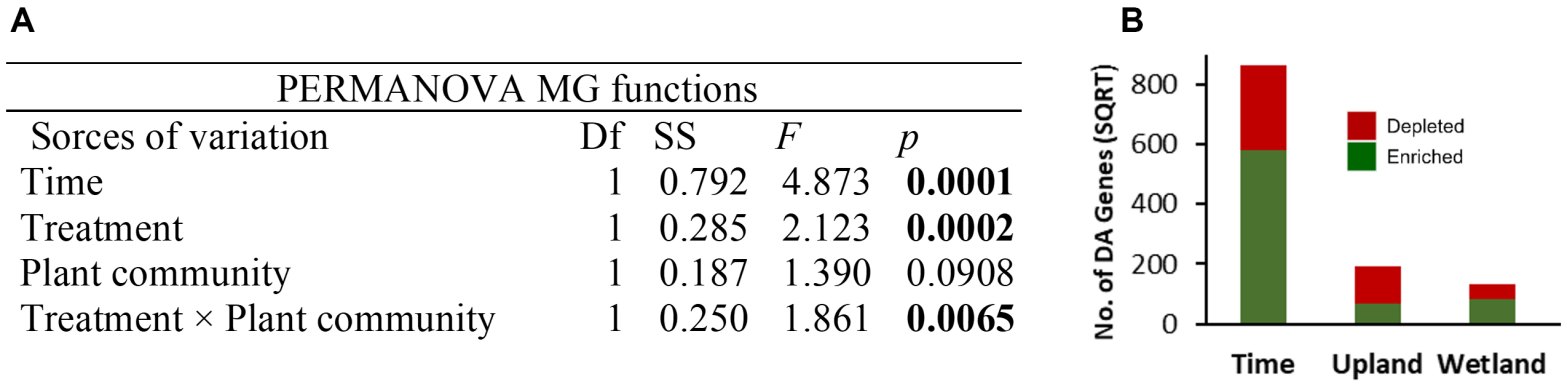
Permutational multivariate analysis of variance (PERMANOVA, 9,999 permutations) on Bray–Curtis dissimilarity matrix based on shotgun metagenomic data after normalization of gene abundance table using the geometric mean of pairwise ratios (GMPR) method **(A)**. The total number of differentially abundant (DA) genes are shown for each group comparison between different time points (May 2019 vs. Oct 2020) and caping treatments (30 cm vs. control) of upland and wetland community **(B)**, details of the DA genes are provided in Figure 7. PERMANOVA, permutational multivariate analysis of variance.

**Figure 7:**
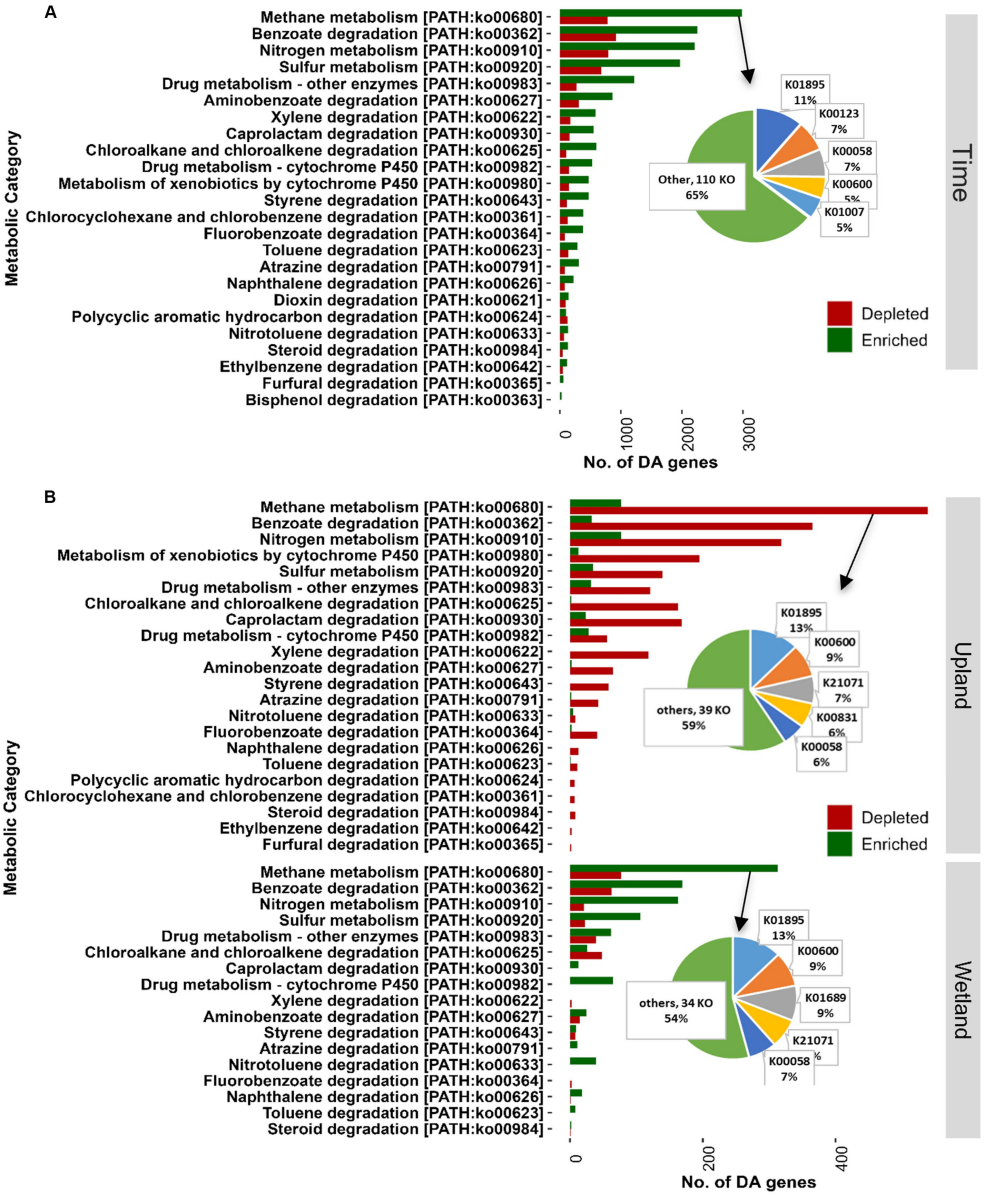
Shotgun metagenomics analysis showing KEGG functional categories for energy metabolism (09102, methane, sulfur, and nitrogen) and xenobiotics biodegradation (09111). Differentially abundant genes were identified between different time points (May 2019 vs. Oct 2020; **A**) and the caping treatments (30 cm vs. control, **B**). Genes were considered as differentially abundant only if FDR-adjusted *p*-values were significant (*p* < 0.05). The list of differentially abundant genes assigned to KEGG Orthology and KEGG functional categories is provided in Supplementary Table S5. 0 cm, control; DA, differentially abundant; FDR, false discovery rate; KEGG, Kyoto Encyclopedia of Genes and Genomes.

Shotgun metagenomics analysis was performed on selected samples (15 samples) from the tailings layer to assess the impact of capping treatments and plant communities on microbial functions. Samples from baseline (May 2019; 3 samples) and GS2 (Oct 2020; 3 samples × 4 treatments) were selected for metagenomic analysis. About 96.5% of reads of quality-controlled shotgun metagenomic data were taxonomically assigned to Bacteria and Archaea and only 0.05% to Fungi, indicating a very low biomass of fungi in the thickened tailings microbiome (Supplementary Table S4). Moreover, 0.07% of reads were assigned to each of Metazoa and Viridiplantae, 0.4% to Viruses, and 2.8% remained unidentified at the kingdom level. Like microbial diversity, the functional profile was significantly changed after two growing seasons compared to baseline samples (Figure 6A). Differential abundance analysis showed that 9% of total genes, mainly including energy metabolism and nutrient cycling, were significantly (log fold change>2, *p* < 0.05) enriched in GS2 samples when compared to baseline samples. However, only 2.2% of total genes were depleted after GS2 (Figure 6B). Further functional analysis specifically focused on the microbial pathways related to energy metabolism (KEGG pathway m09102, methane, sulfur, and nitrogen) and xenobiotics biodegradation and metabolism (m09111). Genes related to methane metabolism, benzoate degradation, nitrogen, and sulfur metabolism were significantly enriched between baseline and GS2 (Figure 7A). Interestingly, similarily to the 16S metabarcoding data, capping influenced the functional profile depending on the plant community (Figure 7B). Indeed, as previously observed for 16S-based alpha diversity and DA of individual taxa, more microbial functions were affected by capping in the upland treatment than in the wetland treatment. Moreover, upland and wetland treatments showed a contrasting response at the functional level (Figure 7B and Supplementary Table S5). Most of the differentially abundant genes belonging to methane metabolism [ko00680], benzoate degradation [ko00362], metabolism of xenobiotics by cytochrome P450 [ko00980], sulfur and nitrogen metabolism pathways [ko00920, ko00910] were found enriched by capping in wetland treatment. In contrast, most of the genes belonging to the above-stated pathways were depleted by capping in upland treatments. In methane metabolism pathways, acetyl-CoA synthetase (K01895; *ACSS1_2*, *acs*) was the most abundant enzyme (Figure 7 and Supplementary Table S5), which is involved in carbon metabolism and methanogenesis (acetate to methane). The second most abundant enzyme was glycine hydroxymethyltransferase (K00600; *glyA*, *SHMT*; glycine hydroxymethyltransferase) which converts formaldehyde (the product of methane oxidation) to C2 or C3 for energy source and assimilation for biosynthesis. In benzoate degradation, KEGG Orthology (KO) K00626 (*ACAT*, *atoB*; acetyl-CoA C-acetyltransferase) and K07516 (*fadN*; 3-hydroxyacyl-CoA dehydrogenase) involved in the conversion of benzoyl-CoA to acetyl-CoA were abundant. In Sulfur metabolism, genes belonging to K01011 (*TST*, *MPST*, *sseA*; thiosulfate/3-mercaptopyruvate sulfurtransferase) were dominant. Moreover, genes related to sulfur oxidation (*dsrABC*, *aprAB*, and *sat*), and pathways for thiosulfate oxidation (*soxABCXYZ* genes) were also found to be differentially affected by time and capping treatments. For nitrogen metabolism, genes encoding for glutamine synthetase, nitrate reductase, including dissimilatory nitrate reduction (K00370; *narG*, *narZ*, *nxrA*) and nitrogen fixation (*nifDKH*) were enriched by time and wetland capping treatments (Supplementary Table S5).

## 4. Discussion

In the present study, prokaryotic and fungal communities were monitored for two growing seasons (GS) in columns composed of thickened oil sands tailings (TT) without capping (0 cm) or with a thin layer of PMM capping (10 or 30 cm) and planted with one of two distinct native boreal plant communities (upland or wetland). Bacterial diversity greatly increased after planting compared to baseline, with a more significant effect in GS1 than in GS2. Likewise, several microbial taxa as well as critical microbial functions in the tailings were significantly enriched after two growing seasons. These changes correlated with the aboveground plant growth, which increased over time (Supplementary Figure S3). The variations in microbial diversity and functions with time were expected as the establishment of plants and the increase in vegetation cover lead to an increase in root biomass and to rhizodeposition-driven changes in soil chemical, physical, and biological properties (Masse et al., 2017; Mohapatra et al., 2021). Most of the bacterial taxa that were enriched over time were potential sulfur-oxidizing bacteria (SOB), ammonia-oxidizing bacteria (AOB), and heterotrophic microorganisms which can contribute to nutrient cycling, plant litter decomposition, and xenobiotic degradation in soils (Mohapatra et al., 2021). Of note, an increase in the relative abundances of Acidobacteria, Actinobacteriota, Chloroflexi, and Planctomycetota was observed over time in tailings when compared to baseline samples. These taxa are among the most abundant bacterial phyla across soil habitats, which positively correlate with the mineralization of organic matter and nutrient cycling (Fierer et al., 2010; Delgado-Baquerizo et al., 2018; Heo et al., 2020). The increase in plant growth and resulting carbon input and nutrient turnover in soil likely influenced the tailings microbial community, favoring taxa more adapted to conditions created by vegetation establishment (Rousk et al., 2010a; Semchenko et al., 2022). Importantly, the uncultured bacteria *Acidithiobacillaceae* KCM-B-112 was significantly increased after two growing seasons when compared to baseline samples. Another study found *Acidithiobacillaceae* KCM-B-112 as abundant taxa in oil-contaminated soil during phytoremediation with poplars (Lopez-Echartea et al., 2020). Here, the significant enrichment of such sulfur-oxidizing acidophilic taxa of the *Acidithiobacillaceae* family and other SOB like *Sulfuritalea* suggests acidification of the alkaline tailings (initial pH of 8.7) over time. In fact, it has been shown that *Acidithiobacillus thiooxidans* decreased the pH in alkaline dolomitic tailings, which further increased nutrient solubilization, especially for phosphorus (Dhakar et al., 2015). On the other hand, typical tailings-associated bacteria (Wilson et al., 2016), particularly *Burkholderiales* (*Comamonadaceae*, *Hydrogenophilaceae*) were outcompeted over time. Similarly, a study found *Comamonadaceae* in young barren soil, which decreased over time with soil age and was completely depleted after about 20 years (Nemergut et al., 2007). In general, *Polaromonas* is found in association with unvegetated soil due to its metabolic versatility and capability to survive in a dormant state (Darcy et al., 2011).

The presence of a PMM cap mainly influenced bacterial alpha diversity and evenness (not richness), beta diversity, and the relative abundance of several dominant bacterial taxa depending upon vegetation type, with a generally limited impact of the thickness of the capping layer (10 cm vs. 30 cm). PMM cover can create micro-level conditions which can impact plant growth, microbial interactions as well as soil properties in the underlying tailings layer, resulting in the enrichment or depletion of specific taxa and functions (Jamro et al., 2014; Duan et al., 2015; Samad et al., 2022). As an example, capping could favor the growth in tailings of some opportunistic taxa that can metabolize the refractory carbon or other nutrients provided by the PMM layer. PMM capping can also alter microbial growth due to changes in oxygen supplies, moisture, pH, pore and particle size, and nutrient fluxes compared to the underlying tailings layer, all of which have been shown to influence the bacterial community composition in soils (Rousk et al., 2010a; Khodadad et al., 2011; Gschwend et al., 2021). Here, the observed changes in microbial communities due to capping were primarily related to changes in the relative contribution of specific bacterial taxa and were mostly observed in the upland plant community. For instance, abundant taxa assigned to *Thiobacillus* and *Nitrosomonadaceae* involved in sulfur and/or nitrogen cycling were enriched in tailings with a PMM cap. *Thiobacillus* (enriched with capping in upland treatments) is a neutrophilic bacteria that can oxidize sulfur in oxic environment through a sulfur-oxidizing multienzyme system (Sox) and in semi-oxic or anoxic environments through the reverse dissimilatory sulfite reductase (rDSR) pathways (Whaley-Martin et al., 2023). Higher relative abundances of other organisms involved in the oxidation of reduced compounds, like *Sulfuritalea* and *Hydrogenophaga*, were observed with capping, again mostly in the upland vegetation treatment, possibly due to the generally dryer conditions (Supplementary Figure S2), root-driven transport of O_2_ to tailings layers, the presence of alternative electron acceptor such as nitrate, or the use of oxygen-independent rDSR pathways for sulfur oxidation. In fact, *sox* and *dsr* genes were differentially abundant with capping. Overall, while the mechanisms remain unclear, the enrichment of such a broad diversity of oxidizing microorganisms indicate that capping did not slow down mineral oxidation processes in the tailing layer.

Metagenomics analysis of key biogeochemical processes, including carbon, nitrogen, and sulfur cycling, showed that the dynamics of energy utilization and transformation also changed with time and, to a lower extent, with capping treatment depending upon the vegetation type. Several genes associated with important soil functions were enriched over time, which can be linked to the development of a win/win partnership between microbes and plants (Liu et al., 2014; Hannula et al., 2019; Wu et al., 2020). Indeed, vegetation growth can supply energy and resources for heterotrophic microorganisms, therefore influencing metabolic functions (Li et al., 2017), and prior studies have shown that plant species influence the microbial composition and associated functions (Wu et al., 2020; Gagnon et al., 2020a). Interestingly here, while capping in the presence of wetland vegetation increased the energy-related metabolic functions (carbon, nitrogen, and sulfur metabolism), the upland treatments showed a contrasting effect, with most of the energy-related metabolic functions being depleted with capping. Similarly, capping also significantly decreased bacterial evenness in the upland but not in the wetland treatment. Previous studies have shown that evenness rather than richness responded rapidly in different stress conditions (Wittebolle et al., 2009; Yi et al., 2022). When microbial communities are highly uneven or exhibit extreme dominance by one or a few species, their functioning is less resilient to environmental stress (Wittebolle et al., 2009; Junkins et al., 2022). For example, manipulation in microbial evenness revealed that resistance to salinity stress was lower when initial microbial communities were uneven (Wittebolle et al., 2009), which suggests that functionally redundant species can better contribute to the ecosystem functioning under stressful conditions. This indicates that aspects of diversity other than microbial richness can play a critical role in community functional stability and may be of interest for further investigation of the effectiveness of reclamation treatments (Shade et al., 2012).

Fungal communities in tailings were dominated by *Ascomycetes*, with a high proportion of the order *Helotiales*, but also included a basidiomycetous component dominated by *Agaricales*. Based on shotgun metagenomics, fungal biomass was very low in tailings (<0.01% of total sequences), and most of the fungal microbiome (especially in the baseline samples) was unclassifiable below the phylum level, which makes it difficult to infer the role of tailings fungal community. Past surveys using 18S rRNA gene sequencing showed that more than 40% of fungal operational taxonomic units (OTUs) were unclassified below the phylum level in the oil sands environment (Richardson et al., 2020). Nevertheless, in the present study, the fungal richness and relative abundance of several dominant taxa significantly increased with time. Capping suppressed some potential plant pathogenic fungi such as *Plectosphaerella* and *Calophoma* (Raimondo and Carlucci, 2018; Hou et al., 2020) while substantially increasing the relative abundance of the beneficial plant-associated fungi *Serendipta* and *Hyaloscypha* in upland and wetland treatments (Vohník et al., 2016; Fehrer et al., 2019; Bzdyk et al., 2022). *Serendipta* and other beneficial fungal taxa from the *Helotiales* (Jean et al., 2021) and *Sebacinales* (Vohník et al., 2016) orders were also detected in PMM, indicating that capping material can serve as a source of potentially beneficial microorganisms to help vegetation establishment. These fungi may promote plant growth and alleviate environmental stress through enhanced phytohormone synthesis, nutrient cycling and uptake, and contaminant biodegradation (Gonzalez et al., 2018; del Barrio-Duque et al., 2019; Jean et al., 2021). Moreover, *Serendipta* can positively and synergistically interact with bacteria, including *Rhizobium* (also detected in PMM), and positive impacts on host plants of these intimate inter-kingdom associations have been observed (del Barrio-Duque et al., 2019; Mahdi et al., 2022). Such beneficial interactions are likely to increase reclamation success and further support the use of PMM as capping material for tailings.

Different responses of bacterial and fungal communities were observed in the presence of wetland and upland vegetation (Hannula et al., 2019; Gschwend et al., 2021). Earlier investigations have confirmed that tree or plant species can contribute to differences in soil properties and, in turn, microbial communities (Samad et al., 2017; Vuong et al., 2020). Indeed, different plant species can produce various microbial resources through root exudates, root cap sloughing, and root turnover, and can be colonized by distinct populations of more or less diverse microorganisms (Zhang et al., 2017; Gagnon et al., 2020b). The plant’s physiological ability to provide oxygen to the rhizosphere also differs among plant species (Callaway, 1995, 1997), and different vegetation types may therefore affect oxygen availability in the underline layer, which is an important driver of microbial composition and functions (Callaway, 1995; Junkins et al., 2022). Upland and wetland species have heterogeneous distributions of shallow and deep roots and vertical stratification of soil resources that can create finely partitioned niches with different microbial resources (Phillips et al., 2014; Vergani et al., 2017). Therefore, the differences in root architecture and growth can have a contrasting effect on soil resources and microbial assembly (Steinauer et al., 2020). Indeed, plant species and pH have been reported as the main factors influencing alpha diversity in PMM-capped and reconstructed oil sand soils (Masse et al., 2017; Mitter et al., 2017). Additionally, the abundance of wetland species like *Salix bebbiana* was positively correlated with bacterial alpha diversity, while the abundance of an upland species, *Pinus banksiana*, was inversely related to alpha diversity (Masse et al., 2017). A similar trend was observed in this study, where alpha diversity (Shannon) was inversely correlated with *P. banksiana* survival [Pearson’s correlation coefficient (*r*) = −0.90] and growth (*r* = −0.21) but was positively correlated with *Salix bebbiana* growth (*r* = 0.77). Wetland species such as willows have better capability to adapt to oxygen shortage conditions and reconstructed environments and concomitant microbial facilitation in such soil environments (Vartapetian and Jackson, 1997). The compatibility of peat-mineral mix (PMM) capping with upland and wetland vegetation and its impact on belowground physicochemical and biological interactions need to be further explored.

## 5. Conclusion

The results from this study provide valuable insights into the succession patterns of microbial communities following the application of organic capping and plantation in oil sands thickened tailings. Depending on the plant community, the microbial structural and functional features varied across the capping treatments. Noteworthy, wetland capping treatments enhanced critical functions related to nutrient cycling and xenobiotic biodegradation. These results suggest that selecting the right combination of capping material and vegetation type may improve microbially-driven processes during the reclamation of oil sands tailings. In line with plant establishment, soil microbial communities have also undergone important changes over two growing seasons, indicating the importance of tracking microbial communities and associated services over extended periods of time during the management and reclamation of tailings. Still, it is difficult to fully disentangle the effect of capping substrate on underline tailings-associated microbial communities due to the complex interplay between multiple biotic and abiotic factors that can change across a few microns in layered soils with distinct textural differences. Further long-term research is required to gain a more comprehensive understanding of microbial responses in the context of oil sands tailings reclamation, with a particular focus on compartments that are more closely associated with plants, such as the rhizosphere and endosphere. A thorough comprehension of soil–plant-microorganism interactions can assist in determining the optimal conditions for capping placement and plant establishment on mine tailings to develop better reclamation practices in such harsh environments.

## Data availability statement

The datasets presented in this study can be found in online repositories. The names of the repository/repositories and accession number(s) can be found in the article/Supplementary material.

## Author contributions

CM, ASé, and DD contributed to the conception and design of the study. DD conducted greenhouse experiments. ASa contributed to the lab experiments, performed bioinformatics analysis, prepared figures, and wrote the manuscript. M-JM contributed to lab experiments. PG contributed to the bioinformatic analysis of shotgun metagenomic data. All authors contributed to the manuscript’s revision and read and approved the submitted version.

## Funding

Funding for this research came from the Natural Resources Canada Office of Energy Research and Development.

## Conflict of interest

The authors declare that the research was conducted in the absence of any commercial or financial relationships that could be construed as a potential conflict of interest.

## Publisher’s note

All claims expressed in this article are solely those of the authors and do not necessarily represent those of their affiliated organizations, or those of the publisher, the editors and the reviewers. Any product that may be evaluated in this article, or claim that may be made by its manufacturer, is not guaranteed or endorsed by the publisher.

## Footnotes

^1^ https://docs.qiime2.org/2020.2/data-resources/

^2^ http://www.htslib.org/doc/samtools-1.0.html

^3^ ftp://ftp.ncbi.nih.gov/pub/taxonomy/taxdump.tar.gz
