# Supplementary material for: Microbial community structural and functional differentiation in capped thickened oil sands tailings planted with native boreal species
Source: Front Microbiol. 2023 Jul 3;14:1168653. doi: 10.3389/fmicb.2023.1168653 (PMC10350512; doi:10.3389/fmicb.2023.1168653)
Supplement: Supplementary file 2 [file Data_Sheet_1.docx]

Supplementary Material

**Microbial community structural and functional differentiation in capped thickened oil sands tailings planted with native boreal species**

**Abdul Samad^1^, Dani Degenhardt^2^, Armand Séguin^1^, Marie-Josée Morency^1^, Patrick Gagné^1^, Christine Martineau^1*^**

*** Correspondence:** Corresponding Author [christine.martineau@nrcan-rncan.gc.ca](mailto:christine.martineau@nrcan-rncan.gc.ca)

**Supplementary Figures**


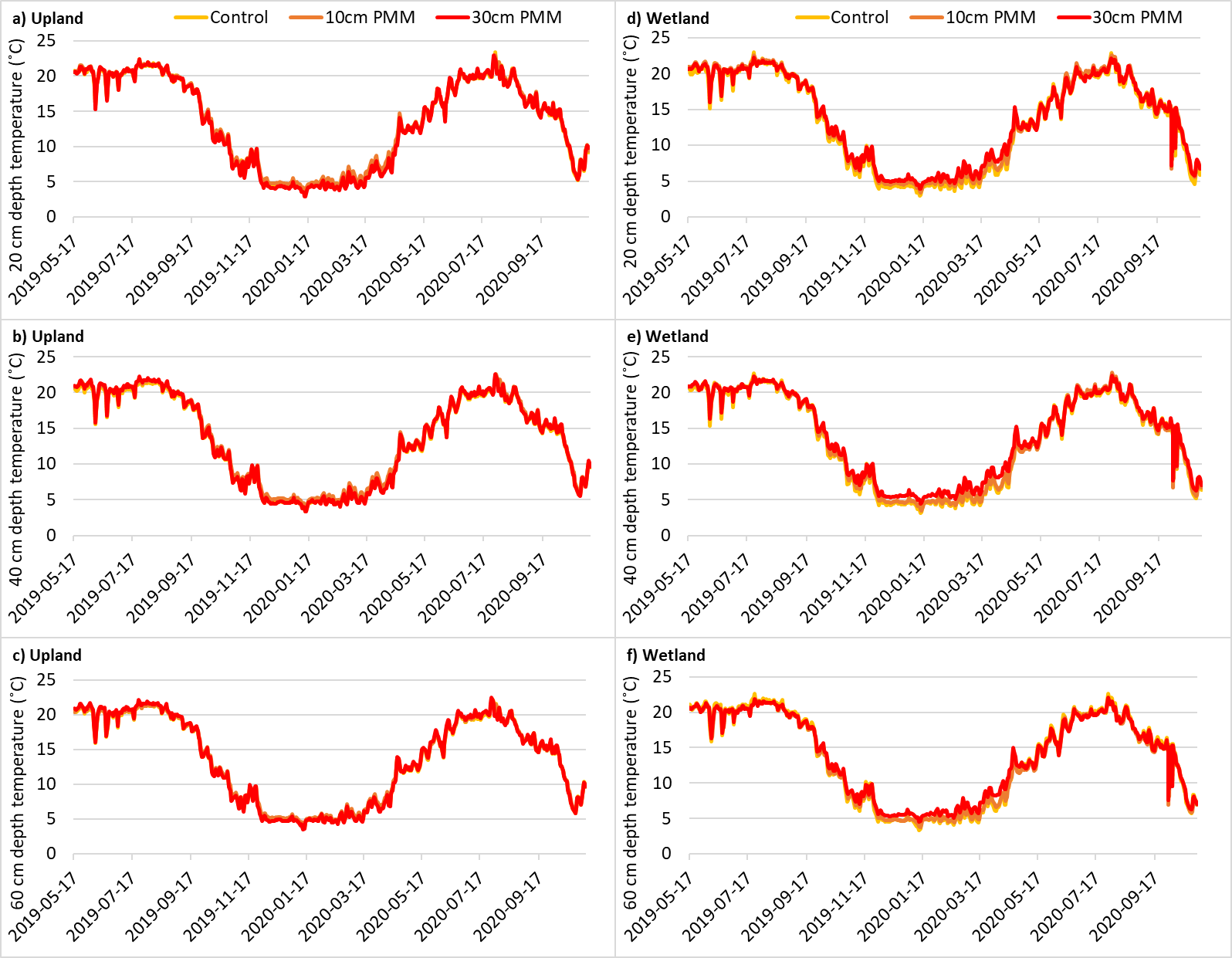


**Figure S1.** Average temperature (n=3) recorded at 20 cm, 40 cm, and 60 cm depths in the thickened tailings upland (a-c) and wetland community (d-f) from May 17th to November 11th, 2020.


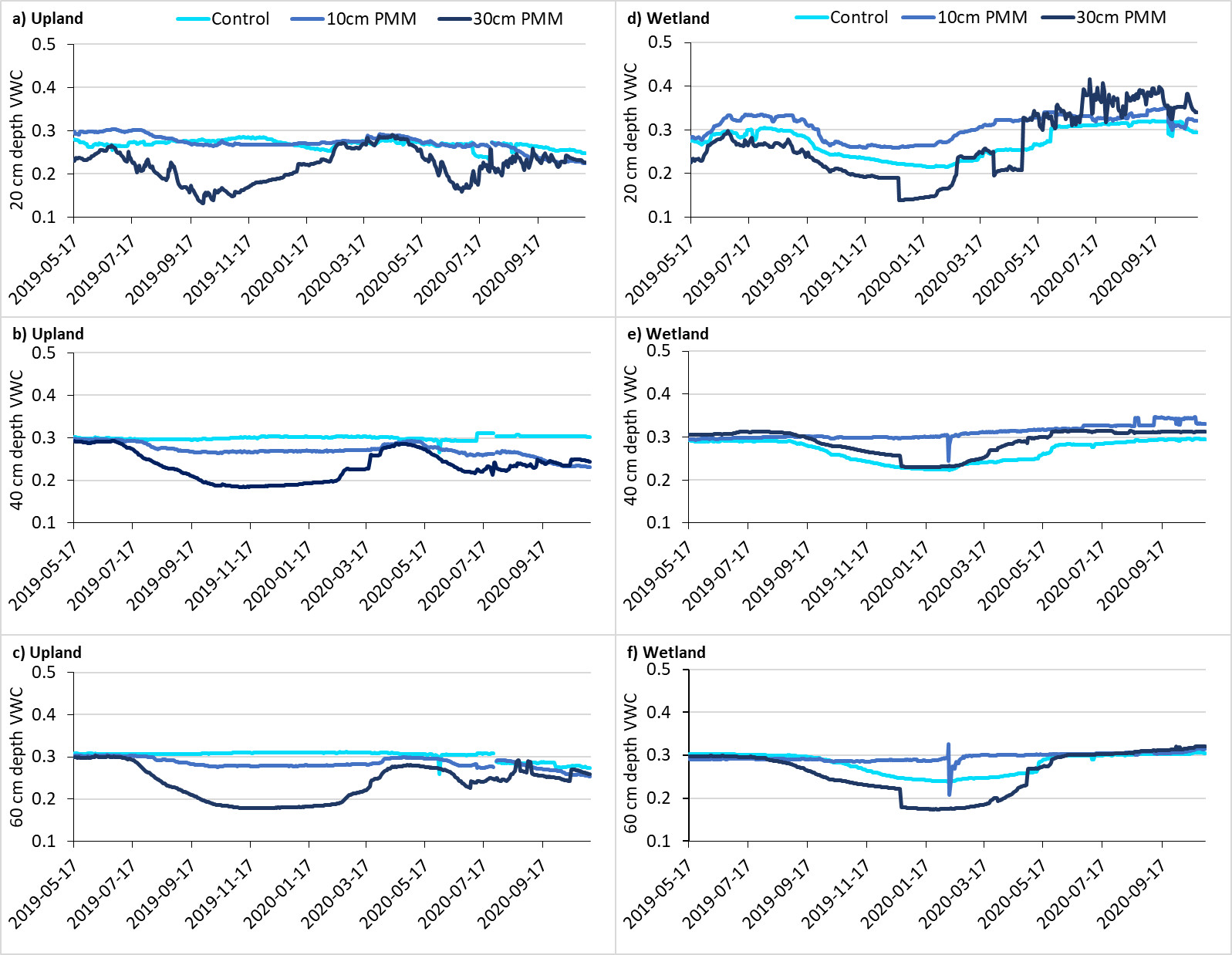


**Figure S2.** Average moisture (n=3) of three treatments at 20 cm, 40 cm, and 60 cm depths in the thickened tailings upland (a-c) and wetland community (d-f) from May 17th to November 11th, 2020. VWC= volumetric water content.

**Figure S3**. Plant survival and growth of woody plants (height and stem diameter) over two growing seasons, A-D for the upland, E-H for the wetland. Welch’s t-test was used to compare plant height between capping treatment and control in each group. Letters indicate significantly different treatments of both years for each species [(abcdef), (ABCDEF), (uvwxyz), (UVWXYZ), (mnopqr)]. Mean ± one standard deviation of the mean (n=4).

**
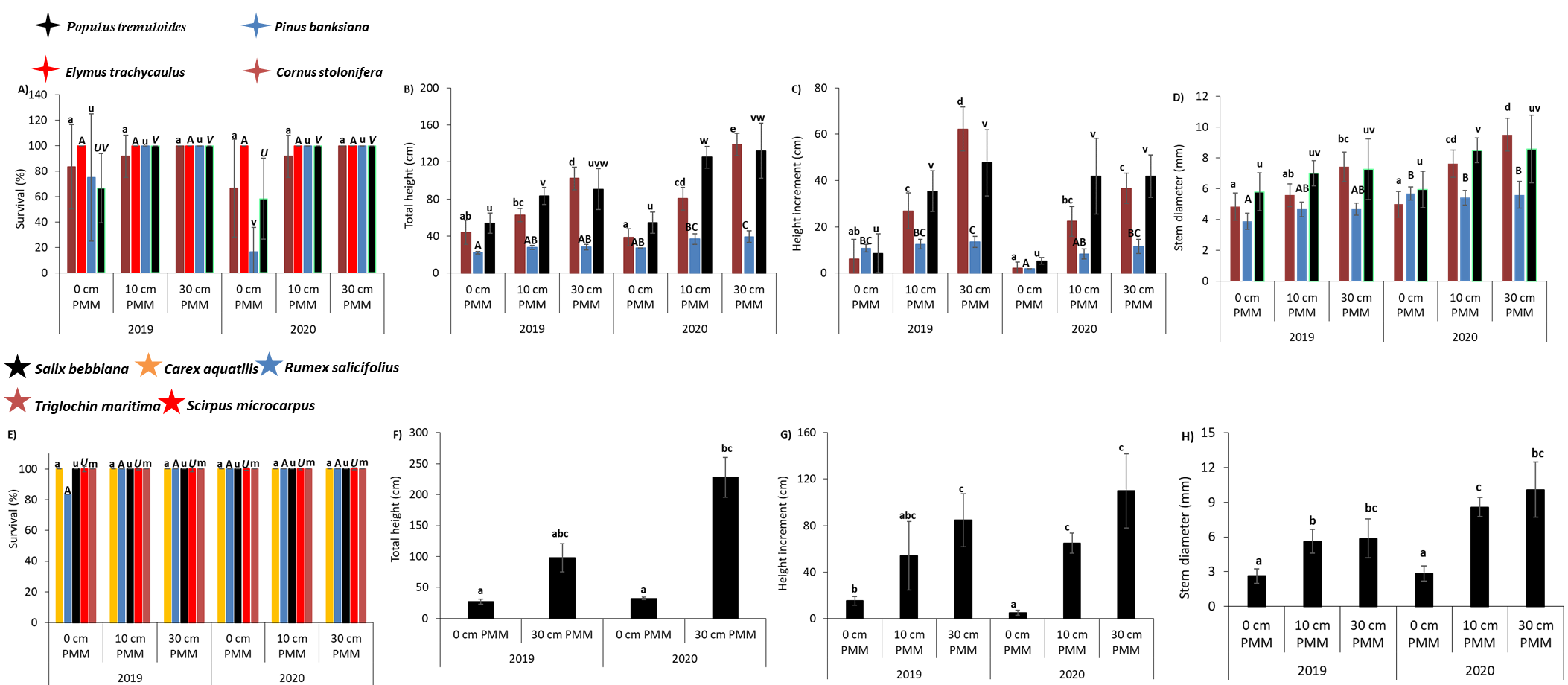
**


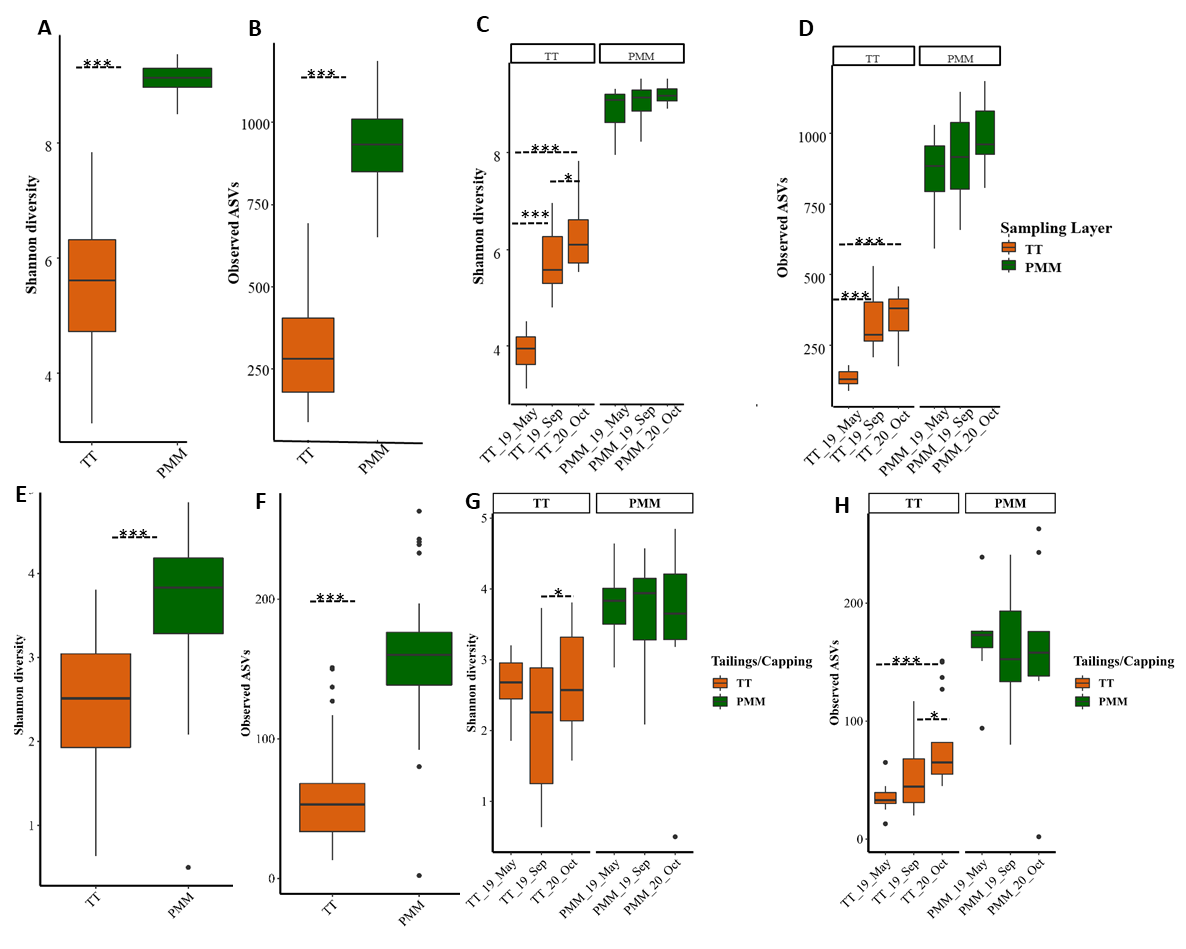


**Figure S4**. Alpha diversity indices of bacterial (A-D) and fungal community (E-H) in thickened tailings (TT) and capping layer (PMM) samples. P-value less than 0.05 is indicated by asterisk, *<0.05, **<0.005, ***<0.0005. 19_May = pre-planting baseline, 19_Sep = GS1, 20_Oct = GS2, PMM = peat mineral mix, GS1= Growth season 1, GS2 = Growth season 2.


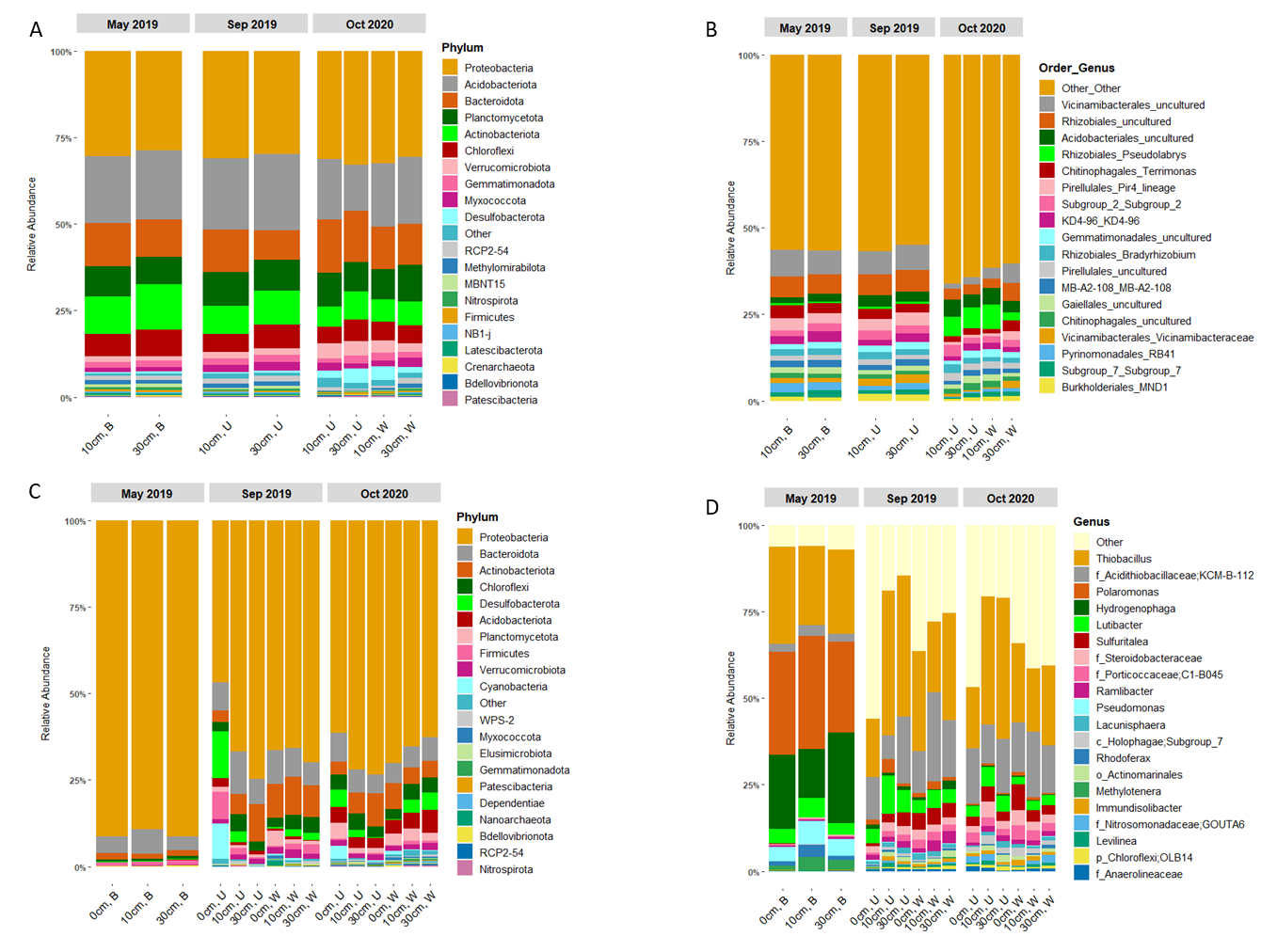


**Figure S5**. Bacterial taxonomic profiles from capping material (PMM; A-B) and thickened tailings (TT; C-D) samples. Only 20 most abundant classified taxa of the bacterial community (phylum and genus level) are shown (n ≥ 3). B = pre-planting baseline, W = wetland, U = upland, 0 cm = control.


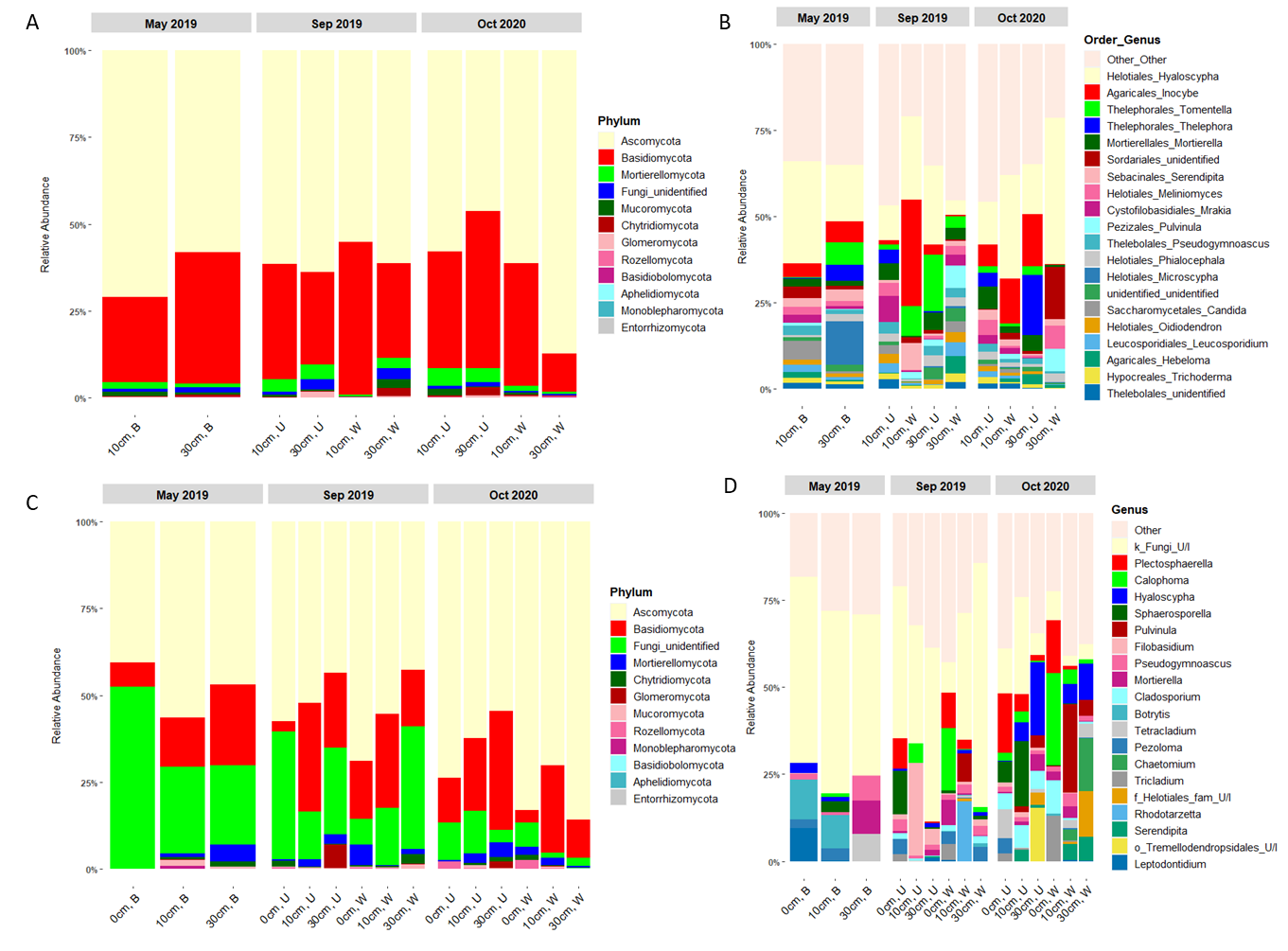


**Figure S6**. Fungal taxonomic profiles from capping material (PMM; A-B) and thickened tailings (TT; C-D) samples. Only 20 most abundant classified taxa of the fungal community (phylum and genus level) are shown (n ≥ 3). B = pre-planting baseline, W = wetland, U = upland, 0 cm = control.

| **Table S1.** The shotgun metagenomic reads which passed each quality control step during bioinformatics data processing. | | | | | | | | | | | | |
| --- | --- | --- | --- | --- | --- | --- | --- | --- | --- | --- | --- | --- |
| Sample name | Capping | Plant community | Sampling time | Raw fragments | Surviving fragments | Surviving fragments % | Surviving single | Total reads | Mapped reads | Mapped % | Properly paired reads | Properly paired % |
| 49m | 0 cm/Control | NA | 2019 May | 1590079 | 1506532 | 0.94 | 44588 | 2899006 | 2792233 | 0.96 | 2568144 | 0.88 |
| 50m | 0 cm/Control | NA | 2020 May | 15066044 | 14227421 | 0.94 | 485928 | 27511180 | 26246858 | 0.95 | 23998774 | 0.87 |
| 51m | 0 cm/Control | NA | 2019 May | 13340255 | 12591646 | 0.94 | 445104 | 24416416 | 23584692 | 0.96 | 21769382 | 0.89 |
| 71f | 30 cm | upland | 2020 Oct | 13080509 | 12236094 | 0.93 | 518548 | 23917742 | 20510788 | 0.85 | 17838766 | 0.74 |
| 73f | 30 cm | upland | 2020 Oct | 7499585 | 7005357 | 0.93 | 303370 | 13710518 | 11396665 | 0.83 | 9899592 | 0.72 |
| 75f | 30 cm | upland | 2020 Oct | 8517355 | 8063131 | 0.94 | 236760 | 15746416 | 13694692 | 0.86 | 12067190 | 0.76 |
| 76f | 0 cm/Control | upland | 2020 Oct | 9532262 | 8967272 | 0.94 | 335444 | 17469704 | 15298424 | 0.87 | 13529788 | 0.77 |
| 77f | 0 cm/Control | upland | 2020 Oct | 11541328 | 11038955 | 0.95 | 143624 | 21516718 | 18758633 | 0.87 | 16942268 | 0.78 |
| 78f | 0 cm/Control | upland | 2020 Oct | 7666240 | 7151092 | 0.93 | 336614 | 14044114 | 11572377 | 0.82 | 9917736 | 0.7 |
| 86f | 30 cm | Wetland | 2020 Oct | 8357421 | 7848470 | 0.93 | 311757 | 15370852 | 10985571 | 0.71 | 9473352 | 0.61 |
| 88f | 30 cm | Wetland | 2020 Oct | 11110631 | 10454417 | 0.94 | 372502 | 20330382 | 13071633 | 0.64 | 11091566 | 0.54 |
| 90f | 30 cm | Wetland | 2020 Oct | 5278955 | 4959773 | 0.93 | 178357 | 9716428 | 5869174 | 0.6 | 5008456 | 0.51 |
| 91f | 0 cm/Control | Wetland | 2020 Oct | 8265394 | 7712995 | 0.93 | 347763 | 15147548 | 13142458 | 0.86 | 11305858 | 0.74 |
| 92f | 0 cm/Control | Wetland | 2020 Oct | 10685202 | 10115757 | 0.94 | 317040 | 19644694 | 16400720 | 0.83 | 14419684 | 0.73 |
| 93f | 0 cm/Control | Wetland | 2020 Oct | 14818890 | 13915084 | 0.93 | 539467 | 27158332 | 24306764 | 0.89 | 21408026 | 0.78 |

**Table S2.** Impact of treatments on bacterial and fungal alpha diversity in thickened tailings samples. Generalized least squares (gls) models and three-way ANOVA were applied to test for the effect post-planting time points (Sep 2019, Oct 2020), capping treatments, plant communities and their interaction on alpha diversity indices (n =3).

| **Shannon** | 16S-Bacteria | | | | ITS2-Fungi | | |
| --- | --- | --- | --- | --- | --- | --- | --- |
| Sources of variation | Df | SS | *F* | *p* | SS | *F* | *p* |
| Time | 1 | 0.944 | 7.884 | **0.010** | 3.491 | 6.577 | **0.017** |
| Capping | 2 | 0.620 | 2.589 | 0.096 | 6.254 | 5.892 | **0.008** |
| Plant community | 1 | 0.475 | 3.970 | 0.058 | 1.974 | 3.718 | 0.066 |
| Capping × Plant community | 2 | 0.908 | 3.792 | **0.037** | 0.191 | 0.180 | 0.836 |
| **Observed ASVs** |  |  |  |  |  |  |  |
| Time | 1 | 13378.778 | 2.575 | 0.122 | 9280.11 | 5.83 | **0.023** |
| Capping | 2 | 14816.889 | 1.426 | 0.260 | 1588.72 | 0.50 | 0.613 |
| Plant community | 1 | 2988.444 | 0.575 | 0.456 | 5329.00 | 3.35 | 0.079 |
| Capping × Plant community | 2 | 29388.222 | 2.828 | 0.079 | 4977.17 | 1.56 | 0.230 |
| **Evenness** |  |  |  |  |  |  |  |
| Time | 1 | 0.016 | 9.051 | **0.006** | 0.075 | 4.020 | 0.056 |
| Capping | 2 | 0.010 | 2.800 | 0.081 | 0.272 | 7.277 | **0.003** |
| Plant community | 1 | 0.011 | 6.157 | **0.020** | 0.037 | 1.962 | 0.174 |
| Capping × Plant community | 2 | 0.016 | 4.581 | **0.021** | 0.010 | 0.258 | 0.775 |

Values in bold indicate statistical significance with FDR corrected p-value less than 0.05.

**Table S3.** Impact of treatments on bacterial and fungal beta diversity of peat mineral mix (PMM) samples. Permutational multivariate analysis of variance (PERMANOVA) on Bray-Curtis dissimilarity matrix (9999 permutations) was used to test for the effect of capping depth (10 cm, 30 cm), plant communities (upland, wetland) and post-planting time points (Sep 2019, Oct 2020) and their interaction beta diversity (n ≥ 3). Geometric mean of pairwise ratios (GMPR) method (Chen et al., 2018) was used for data normalization (fungal and bacterial phylotypes, ASV tables). FDR-adjusted p-value less than 0.05 was considered statistically significant.

|  | 16S-Bacteria | | | | ITS2-Fungi | | |
| --- | --- | --- | --- | --- | --- | --- | --- |
| Sources of variation | Df | SS | *F* | *p* | SS | *F* | *p* |
| Time | 1 | 0.313 | 1.140 | 0.2836 | 0.529 | 1.332 | 0.097 |
| Capping depth | 1 | 0.528 | 1.926 | 0.0838 | 0.347 | 0.874 | 0.6664 |
| Plant community | 1 | 0.213 | 0.778 | 0.5796 | 0.499 | 1.255 | 0.1388 |
| Capping × Plant community | 1 | 0.207 | 0.755 | 0.6028 | 0.368 | 0.927 | 0.5395 |

PMM = peat mineral mix, May 2019 = pre-planting baseline, Sep 2019 = GS1, Oct 2020 = GS2, SS=sum of squares, F= pseudo-F value, *p*= FDR corrected p-value, FDR= false discovery rate, GS1= Growth season 1, GS2 = Growth season 2.

**Table S4.** Read distribution of quality-controlled shotgun metagenomic data at kingdom (k) level.

| Taxon | Percent Abundance |
| --- | --- |
| k__Bacteria | 89.088 |
| k__Archaea | 7.521 |
| k__Other | 2.773 |
| k__Viruses | 0.4102 |
| k__Viridiplantae | 0.0763 |
| k__Metazoa | 0.075 |
| k__Fungi | 0.054 |

**Table S5** (provided in a separate Excel file). Shotgun metagenomics functional analysis. Counts of differentially abundant genes identified between different time points (May 2019 vs. Oct 2020; A) and between camping treatments (30 cm vs. control) for the upland (B) and wetland (C) communities using the DESeq2 package in R. The total number of enriched or depleted genes are shown for each group comparison. Genes for KEGG database functional categories used in global pathway maps and genome maps for energy metabolism (09102, methane, sulfur, and nitrogen) and xenobiotics biodegradation (09111) are shown. We considered the genes as differentially abundant only if FDR-adjusted p-values were significant (p <0.05). The list of differential abundant genes assigned to KEGG Orthology and KEGG functional categories is provided. 0 cm = control, DA = differential abundant, FDR = false discovery rate, KEGG = Kyoto Encyclopedia of Genes and Genome
